# Repression of a large number of genes requires interplay between homologous recombination and HIRA

**DOI:** 10.1093/nar/gkab027

**Published:** 2021-01-28

**Authors:** Ivana Misova, Alexandra Pitelova, Jaroslav Budis, Juraj Gazdarica, Tatiana Sedlackova, Anna Jordakova, Zsigmond Benko, Maria Smondrkova, Nina Mayerova, Karoline Pichlerova, Lucia Strieskova, Martin Prevorovsky, Juraj Gregan, Lubos Cipak, Tomas Szemes, Silvia Bagelova Polakova

**Affiliations:** Institute of Animal Biochemistry and Genetics, Centre of Biosciences, Slovak Academy of Sciences, 840 05 Bratislava, Slovakia; Institute of Animal Biochemistry and Genetics, Centre of Biosciences, Slovak Academy of Sciences, 840 05 Bratislava, Slovakia; Comenius University Science Park, 841 04 Bratislava, Slovakia; Geneton Ltd., 841 04 Bratislava, Slovakia; Slovak Centre of Scientific and Technical Information, 811 04 Bratislava, Slovakia; Geneton Ltd., 841 04 Bratislava, Slovakia; Slovak Centre of Scientific and Technical Information, 811 04 Bratislava, Slovakia; Department of Molecular Biology, Faculty of Natural Sciences, Comenius University in Bratislava, 841 04 Bratislava, Slovakia; Comenius University Science Park, 841 04 Bratislava, Slovakia; Geneton Ltd., 841 04 Bratislava, Slovakia; Department of Cell Biology, Faculty of Science, Charles University, 128 00 Praha 2, Czechia; Institute of Animal Biochemistry and Genetics, Centre of Biosciences, Slovak Academy of Sciences, 840 05 Bratislava, Slovakia; Department of Molecular Biotechnology and Microbiology, Faculty of Science and Technology, University of Debrecen, H-4010 Debrecen, Hungary; Department of Genetics, Faculty of Natural Sciences, Comenius University in Bratislava, 841 04 Bratislava, Slovakia; Department of Genetics, Faculty of Natural Sciences, Comenius University in Bratislava, 841 04 Bratislava, Slovakia; Institute of Animal Biochemistry and Genetics, Centre of Biosciences, Slovak Academy of Sciences, 840 05 Bratislava, Slovakia; Comenius University Science Park, 841 04 Bratislava, Slovakia; Geneton Ltd., 841 04 Bratislava, Slovakia; Department of Cell Biology, Faculty of Science, Charles University, 128 00 Praha 2, Czechia; Advanced Microscopy Facility, VBCF and Department of Chromosome Biology, Max Perutz Labs, University of Vienna, Vienna Biocenter (VBC), 1030 Vienna, Austria; Cancer Research Institute, Biomedical Research Center, Slovak Academy of Sciences, 845 05 Bratislava, Slovakia; Comenius University Science Park, 841 04 Bratislava, Slovakia; Geneton Ltd., 841 04 Bratislava, Slovakia; Slovak Centre of Scientific and Technical Information, 811 04 Bratislava, Slovakia; Department of Molecular Biology, Faculty of Natural Sciences, Comenius University in Bratislava, 841 04 Bratislava, Slovakia; Institute of Animal Biochemistry and Genetics, Centre of Biosciences, Slovak Academy of Sciences, 840 05 Bratislava, Slovakia; Department of Genetics, Faculty of Natural Sciences, Comenius University in Bratislava, 841 04 Bratislava, Slovakia

## Abstract

During homologous recombination, Dbl2 protein is required for localisation of Fbh1, an F-box helicase that efficiently dismantles Rad51–DNA filaments. RNA-seq analysis of *dbl2Δ* transcriptome showed that the *dbl2* deletion results in upregulation of more than 500 loci in *Schizosaccharomyces pombe*. Compared with the loci with no change in expression, the misregulated loci in *dbl2Δ* are closer to long terminal and long tandem repeats. Furthermore, the misregulated loci overlap with antisense transcripts, retrotransposons, meiotic genes and genes located in subtelomeric regions. A comparison of the expression profiles revealed that Dbl2 represses the same type of genes as the HIRA histone chaperone complex. Although *dbl2* deletion does not alleviate centromeric or telomeric silencing, it suppresses the silencing defect at the outer centromere caused by deletion of *hip1* and *slm9* genes encoding subunits of the HIRA complex. Moreover, our analyses revealed that cells lacking *dbl2* show a slight increase of nucleosomes at transcription start sites and increased levels of methylated histone H3 (H3K9me2) at centromeres, subtelomeres, rDNA regions and long terminal repeats. Finally, we show that other proteins involved in homologous recombination, such as Fbh1, Rad51, Mus81 and Rad54, participate in the same gene repression pathway.

## INTRODUCTION

DNA double-strand breaks (DSBs) occur intrinsically during normal cell growth or are caused by exogenous factors. In addition, DSBs are essential intermediates during programmed recombination events, such as meiosis and mating-type switching in yeast (1). Eukaryotic cells have evolved two mechanistically distinct pathways to repair DSBs during mitosis: non-homologous end joining (NHEJ) and homologous recombination (HR) (2). In the NHEJ pathway, which can function throughout the entire cell cycle, two ends of DSBs are ligated together with little or no requirement for homology (3). On the other hand, HR is restricted to the late S and G2 phases, where it uses homologous double-stranded DNA (dsDNA) to mediate error-free DSB repair. HR repair is initiated by the 5′→3′ resection of DSB ends to expose 3′ single-stranded DNA (ssDNA) for replication protein A (RPA). The subsequent displacement of RPA by Rad51 requires HR mediators, such as Rad52 and the Rad55–Rad57 complex in yeast (4–8). The resulting Rad51–ssDNA presynaptic filament is responsible for locating a homologous DNA sequence and then catalysing strand invasion, which pairs the ssDNA overhang with the homologous duplex (9,10). Other proteins, such as the F-box DNA helicase Fbh1 in *Schizosaccharomyces pombe* and the Srs2 helicase in *Saccharomyces cerevisiae*, are negative regulators of Rad51 (11–14). The recruitment of Fbh1 to DSBs is further regulated by the Dbl2 protein (15), which was first identified in a screening as a protein that localises to DNA DSBs (16). The Swi2/Snf2-related Rad54 protein plays multiple roles in regulating Rad51. Based on results from yeast, it serves as a positive regulator at early stages of recombination by enhancing the homologous DNA pairing reaction (17–20), promoting Rad51-mediated strand invasion (19–21), enabling D-loop formation (22–24), and mediating the migration of the nascent Holliday structure (25). However, it also works as a negative regulator of Rad51 at later stages of recombination by preventing non-specific binding of Rad51 to dsDNA or by removing Rad51 from dsDNA to expose a free 3′-OH primer terminus for DNA synthesis (17,26,27). Once DNA synthesis is initiated, two different routes can be used. In the first, the second end of DSB can be engaged to stabilise the D-loop structure, generating two Holliday junctions (28). In the second, the invading strand can be dismantled from the D-loop by helicases such as Fml1 in *S. pombe* and anneal with the complementary strand and associate with the other end of the DSB (29,30). The Holliday junctions are further processed by resolvases, such as Mus81–Eme1 (31). The second mechanism is preferred during mitosis to avoid potentially harmful events such as loss of heterozygosity.

More recently, HR proteins have been shown to play critical roles in maintaining genome integrity during DNA replication (32–34). Rad51, Rad52 and BRCA2 in mammalian cells safeguard arrested forks from becoming dysfunctional and protect dysfunctional forks from excessive resection, thus allowing their successful merger with converging forks (34–36). When forks lose replicative competence, HR proteins restart forks that likely result in the construction of a new replisome (33,37–40).

The mechanism by which chromatin is disassembled and reassembled during DSB repair has also been intensively studied. In budding yeast, chromatin disassembly in the vicinity of a DSB requires the remodelling complex INO80 and MRX (41,42), which is essential for timely DNA resection (43–45). Nucleosome assembly is regulated by histone chaperones, which bind specific histones to mediate their deposition into chromatin. In human cells, chromatin reassembly after DSB repair requires both the HIRA-mediated replication-independent pathway and the CAF-1-mediated replication-dependent pathway, suggesting that they act in a coordinated manner to accurately re-establish the chromatin structure after DNA repair (46). A previous study in budding yeast showed that the histone chaperone Asf1 is required for chromatin assembly after DSB repair by promoting acetylation of histone H3 on lysine 56 via the histone acetyltransferase Rtt109 (41). Interestingly, a recent study reported specific recruitment of both histone H3 chaperones, Hir and CAF-1, to meiotic DSBs in budding yeast (47). In fission yeast, the loss of the HIRA complex leads to an increased susceptibility to DNA damaging agents; this finding supports the idea that the HIRA complex plays a role in protecting cells against DSBs (48).

In addition to DSB repair, the HIRA complex has been implicated in multiple aspects of transcriptional regulation. In some contexts, HIRA is necessary for transcriptional activation. For example, the induction of specific genes in fission yeast in response to environmental stress is HIRA-dependent (49). HIRA is also required for transcriptional repression. *S. cerevisiae* Hir1 and Hir2 were initially identified as repressors of histone gene expression (50). In fission yeast, HIRA represses expression of heterochromatic regions, subtelomeric genes, Tf2 long terminal repeat (LTR) retrotransposons and their remnants, and it also limits the levels of cryptic intragenic transcripts (48,51–53).

However, the interplay between HIRA and HR in the repression of gene expression has not been reported. Here, we show for the first time that the Dbl2 protein functions in repression of regions located closer to long tandem and long terminal repeats compared with the loci with no change in expression. We find that *dbl2* deletion upregulates a large number of genes, including Tf2 LTR retrotransposons, subtelomeric genes, meiotic genes and antisense transcripts. Interestingly, Dbl2 targets overlap with HIRA targets, and simultaneous deletion of *dbl2* and *hip1* or *slm9* does not lead to cumulative increase in expression from most tested loci. Furthermore, genetic interactions between *dbl2* and HIRA extend to histone modifications, growth at 37°C, and sensitivity to the thiabendazole. Consistently, *dbl2* deletion completely restored the silencing defect of *hip1Δ* and *slm9Δ* at the outer centromere, indicating that Dbl2 might act upstream of HIRA. Finally, we show that other proteins involved in HR, including Fbh1, Rad51, Mus81 and Rad54, participate in repression of the tested Dbl2-regulated genes.

## MATERIALS AND METHODS

### Strains, growth media and general methods

The genotypes of the strains used in this study are listed in Supplementary Table S1. Strains carrying a deletion have been either constructed as described previously (54) or purchased from Bioneer (55) and from the National BioResource Project (NBRP, Japan). Rich YES, minimal EMM2 and sporulation PMG-N media were used to grow and mate *S. pombe* strains (56). If necessary, 0.15 g/l G418, 0.1 g/l nourseothricin, 0.2 g/l hygromycin B, 40 μM camptothecin (CPT) or 15 μg/ml thiabendazole (TBZ) were added. For spot assays, 10-fold serial dilutions of exponential phase cultures were spotted onto media in the presence or absence of drug and incubated for 3 days. *S. pombe* was transformed using the lithium acetate method (54). Yeast two-hybrid analysis was performed as previously described (15).

### *β*-galactosidase assay

All *lacZ* reporters previously constructed (48) were introduced into the *dbl2Δ* background by using standard genetic crosses. Approximately 2.2–2.5 × 10^8^ cells were harvested from cultures grown to OD_595_ = 0.75–0.8 in YES at 30°C, washed with H_2_O and stored at –80°C. The β-galactosidase assay was performed as previously described with modifications in the cell lysis protocol (57). Frozen samples were resuspended in 250 μL of Breaking buffer (100 mM Tris-HCl pH 8.0, 1 mM DTT, 20% glycerol) supplemented with 12.5 μl of 0.1 M PMSF and disrupted using glass beads (3 cycles of 2 min vortexing and 2 min chilling on ice). Additional 250 μl of Breaking buffer was added, the cell extracts were transferred to a new tube and centrifuged at 20 000 g for 15 min at 4°C. The clarified supernatants were stored at –20°C. 200 μl of the supernatants were mixed with 800 μl of Z-buffer (60 mM Na_2_HPO_4_, 40 mM NaH_2_PO_4_, 10 mM KCl, 1 mM MgSO_4_, 50 mM β-mercaptoethanol, 10% glycerol) and pre-incubated for 5 min at 30°C. To initiate the reaction, 200 μl of *o*-nitrophenol-β*-*d-galactoside (ONPG) in Z-buffer (4 mg/ml) was added to the consecutive tubes in 30 s interval to keep the incubation time strictly 90 min at 30°C. The reactions were terminated by addition of 500 μl of 1M Na_2_CO_3_ and the absorbance of the reaction mix was measured at 420 nm (Thermo Scientific Multiskan GO). The Bradford assay was used to determine the protein concentration (58). β-galactosidase activity was measured in extracts of three parallel cultures for each strain and is reported as nmol of *o*-nitrophenol/min/mg of total protein.

### Heterochromatin assay

Silencing in the outer domain of centromeric region was assessed using a strain containing *ade6^+^* marker gene inserted into *otr1R* of centromere 1 (59). A marker gene inserted into this region is subjected to strong silencing, which gives a red colour to colonies grown with limited adenine in the medium. Defective silencing in this region leads to whiter colonies.

### Total RNA isolation and RNA-seq library preparation

Total RNA of wt, *dbl2Δ*, wt + CPT*, dbl2Δ* + CPT, *mus81Δ* and *rad51Δ* was isolated as previously described (60). Four biological replicates grown to the exponential phase (OD_595_ = 0.5–0.55) in YES at 30°C were used for each strain. Total RNA was quantified with a Qubit™ 3.0 Fluorometer using the Qubit RNA HS assay (Thermo Fisher Scientific) and the RNA quality was verified by NanoDrop™ 1000 Spectrophotometer (Thermo Fisher Scientific). Recommended input of 1 μg of total RNA was processed to DNA library preparation according to the Illumina TruSeq Stranded Total RNA protocol. Ribosomal RNA depletion and reverse transcription of the cleaved RNA fragments were performed as described in TruSeq Stranded Total RNA Human/Mouse/Rat preparation kit (Illumina). The quantification of the DNA library was performed with a Qubit™ 3.0 Fluorometer using Qubit DNA HS assay (Thermo Fisher Scientific). Quality of the DNA library was analysed with an Agilent 2100 Bioanalyzer (Agilent Technologies). Sequencing was performed using Illumina NextSeq500 technology with the application of paired-end sequencing (2 × 75 bp reads). Adapters and low-quality ends of sequenced reads were removed using Trimmomatic (version 0.36) (61) based on quality control statistics generated by FastQC (version 0.11.5). After trimming, fragments without sufficient length of both reads (at least 35 bp) were removed from read sets. Filtered reads were used in downstream analyses.

### RNA-seq data analysis

Expression of individual transcripts for each sequenced sample was estimated separately using Salmon (version 0.7.2) (62). Count vectors were aggregated into the summary table and normalised for different sequencing depths among samples using edgeR (version 3.12.1) (63). The tool also assessed the statistical significance of expression changes among biological replicates of selected groups (WT and *dbl2* mutant, normal and CPT environment). We considered transcripts to be significantly changed if they met three conditions: (i) ≥1.5-fold change between two conditions; (ii) a calculated false discovery rate (FDR) ≤0.05 and (iii expression was observed in each biological replicate. Due to high sequence similarity of the Tf2 LTR retrotransposons, we analysed these together by summing all reads mapped to any of the Tf2 location (13 records in the PomBase gff3 annotation). Finally, we excluded transfer RNA and ribosomal RNA due to sequence similarity between paralogues and great deviations induced by rRNA depletion, respectively. Alternatively, filtered reads were also mapped to the reference yeast genome using Hisat2 (version 2.0.5) (64). Quality of the mapping was assessed using summary reports generated by Qualimap (version 2.2.1) (65). Coverage tracks were extracted from resulting BAM files using the bamCoverage tool from DeepTools (version 3.1.3) (66) and visually inspected with IGV (version 2.4.8) (67). Data analysis processing was automated using pipelines implemented in the SnakeLines framework (manuscript in preparation) running on the Snakemake workflow engine (version 5.2.2) (68). Reference genomic and transcriptomic sequences of *S. pombe* were downloaded from the PomBase database (version ASM294v2) (69). We used an associated gff3 annotation file to identify genomic regions with low sequential complexity. In particular, we selected all regions annotated as repeats, retrotransposons, polyA, centromeres, gaps or low complexity regions. We also reannotated the genome with Tandem Repeats Finder (version 4.9.0) (70), which identified 1688 tandem repeat regions between 24 and 9368 bp long (median length 46 bp). According to the length of the repeated motif, we divided them into two groups: short tandem repeats (motif length < 7 bp, 266 regions) and long tandem repeats (motif length > 6 bp, 1422 regions). Finally, we used the TetraplexFinder tool from QuadBase2 (other sources 4) to locate G-quadruplex motifs in the genome (71). Functional enrichment analysis was performed using g:Profiler (version e94_eg41_p11_6f51822) with the g:SCS multiple testing correction method applying significance threshold of 0.05 (72,73).

The significance of overlaps between different gene lists was assessed using the hypergeometric test. We considered the definition of the hypergeometric distribution with *k* successes (upregulated genes in both mutants) in *n* draws (upregulated genes in mutant 1), without replacement, from a finite population of size *N* (all genes) that contains exactly *K* objects with the feature (upregulated genes in mutant 2) that fits our data well. The *P*-value indicates the probability that the observed overlap happened by chance.

### Quantitative PCR (qPCR)

RNA was isolated from yeast strains grown in YES at 30°C either to the exponential phase (OD_595_ = 0.5–0.55) or to the stationary phase (OD_595_ = 7–8) using Thermo Fisher Scientific kit. First strand complementary DNA (cDNA) was prepared using Thermo Fisher Scientific components (OligodT, Random hexamer, RiboLock RNase Inhibitor and RevertAid Reverse Transcriptase) according to manufacturer's instructions. For qPCR, FastStart DNA Master SYBR Green master mix (Roche) was used as instructed. Two genes (*act1* and *tbp1*) were used for normalisation. Primers used for measuring gene expression are listed in Supplementary Table S2.

### MNase digestion of chromatin, library preparation and analysis

One-hundred millilitres of wt (SP65), wt (SP072), *dbl2Δ* (SP067) and *hip1Δ* (SP456) were grown to OD_595_ = 0.75–0.8 in YES at 30°C. MNase digestion of chromatin, DNA sequencing and bioinformatics analyses were performed as previously described (74) with minor modifications. Three biological replicates were used for each sample. Fragmented DNA libraries were prepared using TruSeq Nano DNA Library Prep Kit (Illumina) according to the manufacturer‘s protocol with some modifications. We prepared libraries with half the volume of reagents and omitting the fragmentation step at the beginning of library preparation and without size selection. After the end repair step, all DNA fragments were captured with a fourfold volume of AMPure XP beads (Beckman Coulter). Next, DNA fragments were 3′-adenylated and ligated to indexed adapters using TruSeq DNA CD Indexes (Illumina). Libraries were amplified with eight cycles of PCR. The final DNA libraries were quantified with a Qubit™ 3.0 Fluorometer using the Qubit DNA HS assay (Thermo Fisher Scientific). The quality of the DNA libraries was analysed by Agilent 2100 Bioanalyzer using a High Sensitivity DNA assay (Agilent Technologies). The libraries were normalised to 4 nM and then pooled, denatured and diluted to a loading concentration of 1.8 pM for clustering at high output Illumina flowcell. Sequencing was performed using Illumina NextSeq500 technology with the application of paired-end sequencing (2 × 75 bp reads).

### Chromatin immunoprecipitation and deep sequencing (ChIP-seq)

We performed ChIP-seq analyses with two independent biological replicates for each sample as previously described (75) with the following modifications. Four-hundred millilitres of *S. pombe* WT and *dbl2*Δ cells were grown to the exponential phase (OD_595_ = 0.5) in YES medium at 30°C. The extracted chromatin was sheared with the Bioruptor sonicator (Diagenode) using 30 cycles of 30 s ON, 30 s OFF at high power settings. For each IP, 5 μg of the respective antibody were used (H3: ab1791, H3K9me2: ab1220, both from Abcam). The washed precipitated material and input chromatin extracts were decrosslinked for 6 h at 65°C, treated with RNase A (Thermo Fisher Scientific) for 1 h at 37°C followed by proteinase K (Roche) treatment for 2 h at 55°C. DNA was isolated using phenol–chloroform extraction and sodium acetate/ethanol precipitation. DNA was further purified on SPRIselect beads (Beckman Coulter, B23317) to remove RNA and small DNA fragments.

For library construction, Illumina TruSeq Nano DNA kit with Illumina TruSeq DNA CD Indexes were used according to the manufacturer's instructions, with the following exceptions: no initial DNA fragmentation was performed and DNA was purified after end-repair using 4 volumes of AMPure Beads. Libraries were pooled and sequenced on an Illumina NextSeq 500 instrument (35 nt paired-end mode) using the NextSeq 500/550 High Output Reagent Cartridge v2 75 cycles with the NextSeq 500/550 High OutPut Flow Cell Cartridge v2.5. Adapter sequences were trimmed during export to FASTQ. Reads were then quality-trimmed using Trimmomatic 0.39 (61). The obtained reads were mapped to the fission yeast genome (ftp://ftp.pombase.org/pombe/genome_sequence_and_features/genome_sequence/; downloaded 09/2020) using HISAT 2.1.0 (76) and samtools 1.7 (77). BAM files were further processed using Deeptools 3.3.1 (66). For each sample, a read coverage was determined using bamCoverage. Normalisation of each H3K9me2 coverage dataset to the corresponding total H3 coverage was applied using bigwigCompare. The log2-transformed H3-normalised data from *dbl2*Δ were further normalised to wt. Coverage files were inspected visually in IGV 2.6.3 (67) and analysed using R 4.0.2 (www.r-project.org/) and RStudio 1.2.5019. The scripts used for ChIP-seq data processing and analyses are available at https://github.com/mprevorovsky/bagelova-polakova-dbl2-histones.

### Western blotting analysis

Primary anti-histone H3 (ab176842), anti-histone H3K9ac (ab4441), anti-histone H3K4me3 (ab8580), anti-histone H3K9me2 (ab1220) and anti-histone H3K9me3 (ab8898) antibodies and the secondary goat anti-rabbit IgG H&L (conjugated to horseradish peroxidase, HRP) (ab205718) and goat anti-mouse IgG H&L (HRP) (ab205719) antibodies were purchased from Abcam. All the other chemicals were obtained from Sigma-Aldrich. For each strain, one liter of culture was grown in YES medium to mid-log phase (OD_595_ = 0.8) and the cells were collected by centrifugation (4000 g for 5 min at 4°C). Yeast cell powders were prepared from frozen cell pellets using SPEX SamplePrep 6770 Freezer/Mill cooled by liquid nitrogen. Proteins were extracted using Buffer II (50 mM Tris–HCl pH 8.0, 300 mM NaCl, 1 mM EDTA, 0.1% NP-40, 1 mM Mg-acetate, 1 mM imidazole, 10% glycerol, complete protease and phosphatase inhibitors and 1 mM PMSF) (78) in a ratio of 1 g of yeast powder to 1 ml of Buffer II for 20 min at 4°C. Extracts were cleared by centrifugation (41 000 g for 10 min at 4°C) and proteins were subjected to sodium dodecyl sulphate polyacrylamide gel electrophoresis (SDS-PAGE) and western blotting onto a polyvinylidene fluoride membrane (PVDF, 0.45 μm, Millipore). For the immunodetection, the primary and secondary antibodies were used at dilutions of 1:2000 and 1:10 000, respectively. The quantitative analysis was done on digitalized images using ImageJ software (National Institutes of Health). The Student's *t*-tests for paired comparison were performed on the data from experiments repeated four times.

## RESULTS

### Dbl2 represses expression of both coding and non-coding genes

**Figure 1.**
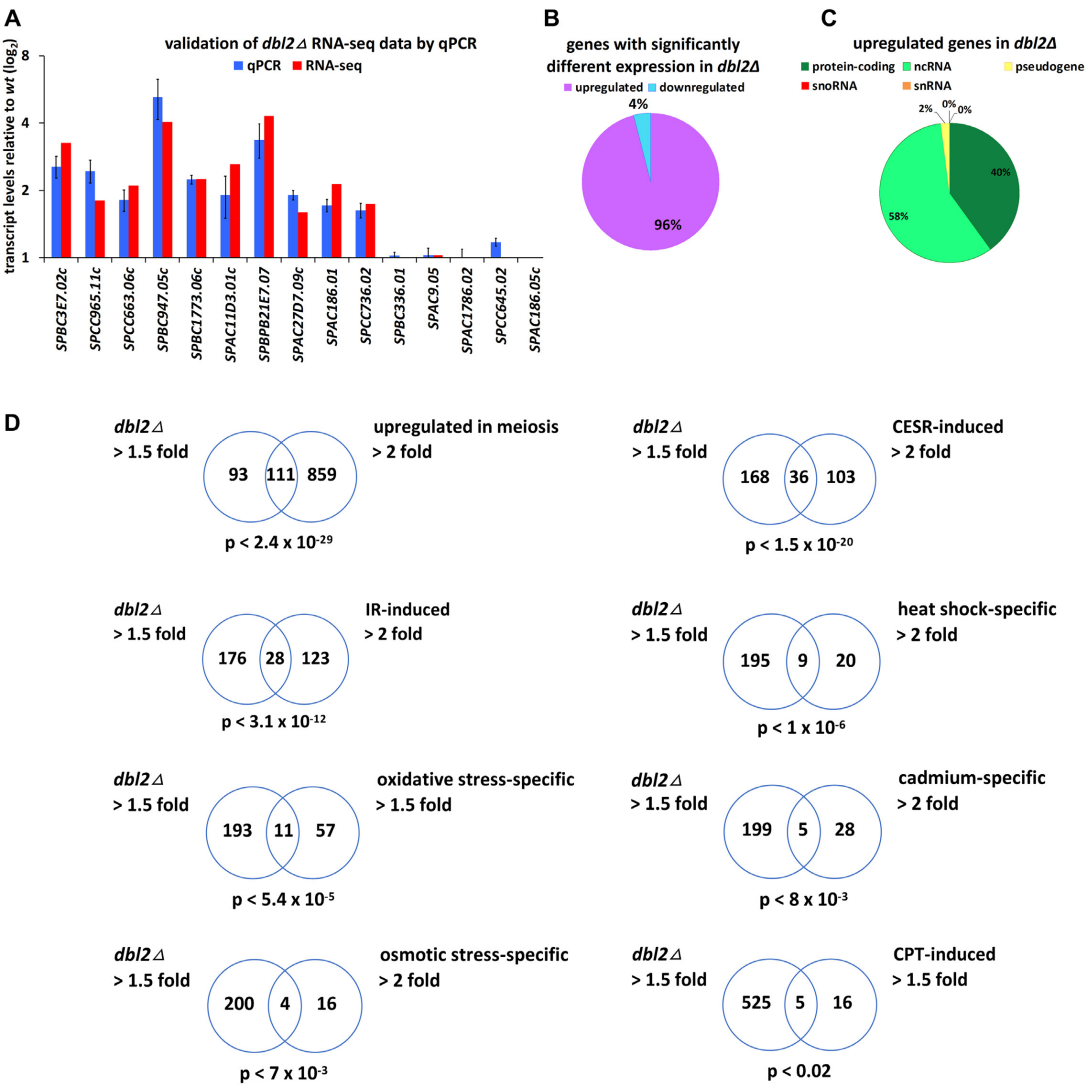
Deletion of *dbl2* leads to significant change in expression of more than 500 genes. A wild-type strain (SP065) and *dbl2Δ* mutant (SP067) were cultivated in standard YES media and their transcriptomes were analysed using RNA-seq. (**A**) *dbl2Δ* RNA-seq data for multiple genes were validated using qPCR. Upregulated genes and genes with no difference in expression were randomly selected from *dbl2Δ* RNA-seq data. The plotted values are the mean of four independent biological replicates ± standard error of the mean. (**B**) The majority of differentially expressed genes in the *dbl2Δ* mutant are upregulated. (**C**) Upregulated genes in the *dbl2Δ* mutant (511) are represented mostly by non-coding (295) and protein-coding genes (204), with a few pseudogenes (10), snoRNA (1) and snRNA (1). (**D**) Venn diagrams showing overlap between genes upregulated in the *dbl2Δ* mutant and genes upregulated under the indicated condition. Only genes included in both analyses were compared. The *P*-value indicates the probability that the observed overlap happened by chance.

Yeast two-hybrid system analysis showed that Dbl2 fused to the GAL4 DNA-binding domain can trigger expression from the reporter gene, suggesting a possible function of Dbl2 in gene expression regulation (Supplementary Figure S1). To address this phenomenon, we analysed the expression profiles of *dbl2Δ* and wild-type cells grown in YES medium to the exponential phase using strand-specific RNA-seq. We validated RNA-seq data for multiple genes using qPCR (Figure 1A). We observed much smaller differences in the level of transcripts of selected genes between *dbl2Δ* and wild-type cells grown in minimal EMM2 medium to the exponential phase (Supplementary Figure S2A) or in cells grown in YES medium to the stationary phase (except for *SPBPB2B2.08*) (Supplementary Figure S2B).

**Figure 2.**
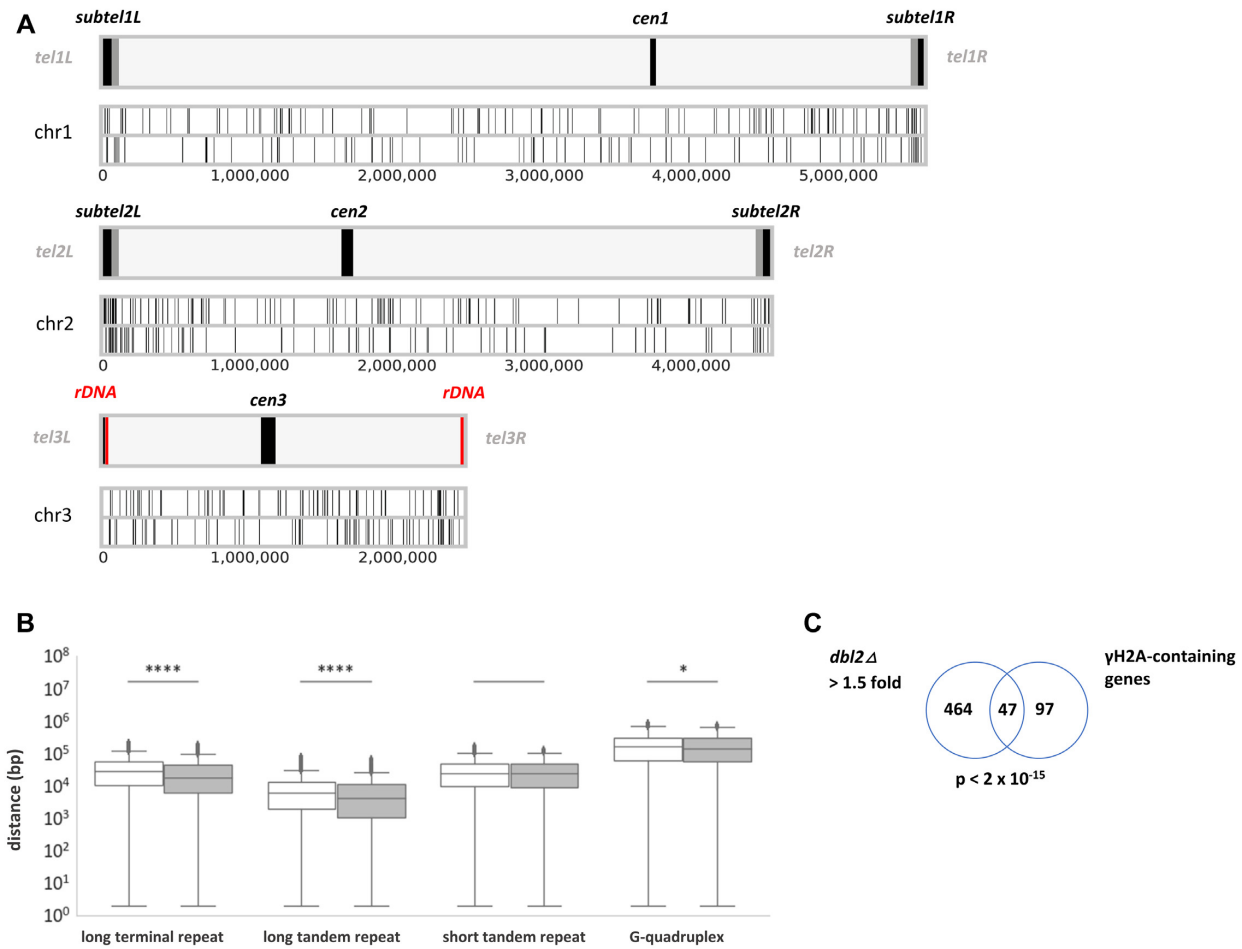
Genome-wide localisation of genes misregulated in *dbl2Δ* for the three *S. pombe* chromosomes. (**A**) Illustration of *S. pombe* chromosomes highlighting the subtelomeres (*subtel*)—box in grey and black adjacent to telomeres. The black box of the subtelomere represents telomere-adjacent *SH* region, which contain heterochromatin and the grey box of the subtelomere represents a highly condensed knob structure generated by Sgo2 (83). The term ‘subtelomere’ refers to the 100-kb-long region from the telomere end of chromosomes 1 and 2 (83). The ends of chromosome 3 contain rDNA repeats marked in red close to the telomeres; rRNA was depleted in the RNA-seq experiment. The length of the three *S. pombe* centromeres *cen1*, *cen2* and *cen3*, is about 40 kb, 65 kb and 110 kb, respectively (153). Differentially expressed (DE) genes between *dbl2Δ* (SP067) and wt (SP065) on forward and reverse strands are represented as vertical lines on the upper and lower part of the chromosomal plot, respectively. DE genes in the *dbl2Δ* mutant were significantly enriched at the subtelomeric regions of chromosomes I and II (78 from 530 DE genes, chi-squared test, *P* < 2.12 × 10^−49^) but not at the centromeric region (4 from 530 DE genes, chi-squared test, *P* < 0.97). The statistics were performed using chi2 contingency test implemented in the *scipy* Python package (154). (**B**) Distance between Dbl2-affected genes and the closest specific genomic element. DE genes between wild-type and the *dbl2Δ* mutant (grey colour) are significantly closer to long terminal repeats and long tandem repeats than genes without significant change in expression (white colour). The lines across the boxes represent the median values of distance for NDE versus DE genes from long terminal repeats—26 577 bp vs 16568 bp; long tandem repeats—6000 bp versus 4114 bp; short tandem repeats—23 987 bp versus 23 030 bp; and G-quadruplexes—153 155 bp versus 139 204 bp. The boxes show the middle 50% of scores while the whiskers represent the maximum and minimum values, except for points determined as outliers outside of the 1.5 × interquartile range. (**C**) Venn diagram showing overlap between genes upregulated (>1.5-fold) in the *dbl2Δ* mutant with genes covered with γH2A. Only genes included in both analyses were compared. The *P*-value indicates the probability that the observed overlap happened by chance.

There were 530 significantly altered RNA levels in the *dbl2Δ* mutant grown in YES medium to the exponential phase (FDR < 0.05; fold change > 1.5), indicating that Dbl2 influences the expression of a large number of loci (Supplementary Table S3). The majority of these genes (96%), including both protein-coding and non-coding genes, showed increased transcript levels in the *dbl2Δ* mutant (Figure 1B). Deletion of *dbl2* increased the expression of at least 4% of fission yeast protein-coding genes. Interestingly, 58% of the upregulated genes belong to non-coding RNA, mainly antisense RNA (Figure 1C). Antisense RNA can arise in the cells either via read-through transcription at convergent genes or via initiation of antisense transcription from cryptic promoters. The antisense RNAs in the *dbl2Δ* mutant did not map preferentially to convergent genes (49%), suggesting that they are not a result of faulty transcriptional termination (Supplementary Table S3). The transcriptome of the *dbl2Δ* mutant was further enriched for transcripts from Tf2 LTR retrotransposons (2.1-fold increase), as well as transcripts from subtelomeric genes (Figure 2A), and meiotic genes (*P* < 2.4 × 10^−29^) (Figure 1D) (79). We analysed expression levels of individual Tf2 LTR retrotransposons in strains with an integrated *lacZ* reporter adjacent to an LTR (48). Reporter gene analysis showed that the suppression of only a few Tf2 mRNA levels is dependent upon Dbl2 (Supplementary Figure S2C).

Furthermore, many of the genes upregulated in the *dbl2Δ* mutant overlap with the core environmental stress response genes (CESR) (*P* < 1.5 × 10^−20^) (80) and less with irradiation-induced (*P* < 3.1 × 10^−12^) (81), heat-shock-specific (*P* < 1 × 10^−6^), oxidative-stress-specific (*P* < 5.4 × 10^−5^), cadmium-specific (*P* < 8 × 10^−3^) and osmotic stress-specific (*P* < 7 × 10^−3^) genes (Figure 1D) (80). The *dbl2Δ* mutant is sensitive to the topoisomerase poison CPT (15), which prevents normal DNA re-ligation and therefore causes DSBs. To determine whether the changes in RNA levels in *dbl2Δ* are due to increased DNA damage, we analysed the expression profile of wild-type cells grown in YES medium supplemented with CPT using RNA-seq (Supplementary Table S3). When we compared the expression response to CPT with the response to *dbl2* deletion, we detected only a very small overlap between the two profiles (*P* < 0.02) (Figure 1D). These results do not support the notion that the altered expression profile in *dbl2Δ* is due to increased DNA damage, although we cannot exclude this possibility. To further analyse the protein-coding genes influenced by Dbl2, we performed functional enrichment analysis. The Gene Ontology (GO) terms that are overrepresented in the list of upregulated protein-coding genes are related to meiosis, detoxification and transport (Supplementary Figure S3).

*S. pombe* possesses three chromosomes, of which chromosomes I and II contain a distinct form of heterochromatin at their ends, whereas chromosome III contains arrays of ribosomal DNA repeats next to the telomeres (82,83). Our analysis of the localisation of misregulated genes in the *dbl2Δ* mutant showed that, while there were many misregulated genes throughout the genome, they were significantly enriched at the subtelomeric regions of chromosomes I and II (chi-squared test, *P* < 2.12 × 10^−49^) (Figure 2A). We did not identify similar enrichment of misregulated genes at the centromeric region (chi-squared test, *P* < 0.97) (Figure 2A). We next compared the distribution of the misregulated loci in *dbl2Δ* with the distribution of repetitive DNA elements such as LTRs (84), motifs of seven or more (long tandem repeats), motifs of six or fewer (short tandem repeats) (70) and G4 structures (71). We found that the localisation of the misregulated genes significantly correlated with the localisation of long terminal and long tandem repeats (*P* < 6.7 × 10^−11^ and *P* < 2 × 10^−6^, respectively) (Figure 2B, Supplementary Figure S4). Nevertheless, we observed only a very small correlation between the transcript fold changes and the distance from a long terminal or a long tandem repeat (Supplementary Figure S5). Furthermore, we did not find any correlation with the localisation of short tandem repeats and G4 structures (Figure 2B).

Closer inspection of the localisation of the misregulated genes revealed that they significantly overlapped with genomic loci decorated by phosphorylated histone H2A (γH2A) (*P* < 2 × 10^−15^) (Figure 2C). γH2A marks are formed at natural replication fork barriers, retrotransposons, heterochromatin region and at the region repressed by Clr3/Clr6-mediated histone deacetylation (85). Overall, these data suggest that Dbl2 represses the expression of meiotic genes, subtelomeric genes, Tf2 LTR retrotransposons and non-coding RNA genes. Compared with the loci with no change in expression, the misregulated loci are closer to long terminal and long tandem repeats.

### Dbl2 represses loci targeted by HIRA

**Figure 3.**
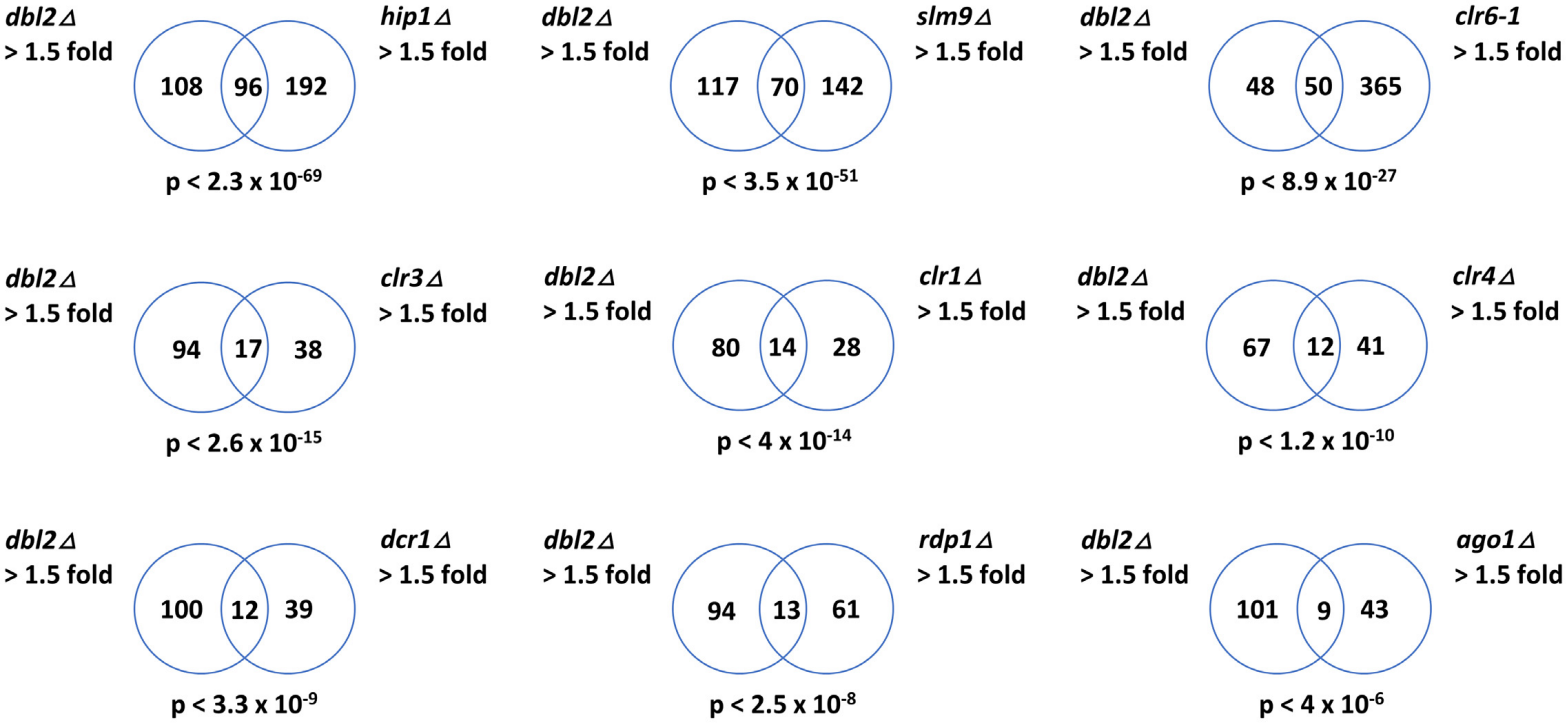
Comparison of upregulated genes in the *dbl2Δ* and selected mutants that are known to affect gene expression. Venn diagrams showing overlap between genes upregulated (>1.5-fold) in the *dbl2Δ* mutant with genes upregulated (>1.5-fold) in the indicated deletion mutant. Only genes included in both analyses were compared. The *P*-values indicate the probability that the observed overlap happened by chance.

In *S. pombe*, the main pathways that have been implicated in the repression of gene expression include RNA interference (RNAi) machinery (*dcr1, ago1, rdp1*) (86–88), silencing machinery (*clr1*, *clr3*, *clr4* and *clr6*) (89–94) and histone chaperone HIRA (*hip1* and *slm9)* (48,95). To determine whether Dbl2 fits into one of these pathways, we compared our *dbl2Δ* RNA-seq data with tiling array data from previous studies (48,92). We observed the most significant overlap between genes upregulated in the *dbl2Δ* mutant and genes upregulated in the *hip1Δ* and *slm9Δ* mutants (*P* < 2.3 × 10^−69^ and *P* < 3.5 × 10^−51^, respectively) and, to a lesser degree, with the *clr6–1* mutant (*P* < 8.9 × 10^−27^) (Figure 3). We identified less significant overlap between genes upregulated in the *dbl2Δ* mutant and genes upregulated in mutants involved in silencing machinery – *clr3Δ* (*P* < 2.6 × 10^−15^)*, clr1Δ* (*P* < 4 × 10^−14^) and *clr4Δ* (*P* < 1.2 × 10^−10^) – and the RNAi pathway – *dcr1Δ* (*P* < 3.3 × 10^−9^), *rdp1Δ* (*P* < 2.5 × 10^−8^) and *ago1Δ* (*P* < 4 × 10^−6^). The greater overlap with the HIRA complex and Clr6 indicates that Dbl2 might participate within the same biological pathway. This overlap cannot be simply explained by downregulation of genes encoding the HIRA complex or Clr6 in *dbl2Δ* because the loss of *dbl2* did not lead to a change in their expression. HIRA histone chaperone mediates replication-independent histones H3-H4 deposition (96–100) and has been implicated in assembly of heterochromatin and silencing in various organisms (101–104). Clr6 functions as the catalytic core of the class I histone deacetylase (HDAC) complex, which regulates gene expression from numerous loci (93). Of note, HIRA plays a role in inhibiting expression of many genes, which are normally repressed in growing vegetative cells (48). These genes include those located in subtelomeric regions, Tf2 LTR retrotransposons, meiotic genes and antisense transcripts (48,51).

### Genetic interactions between *dbl2* and genes associated with gene repression

**Figure 4.**
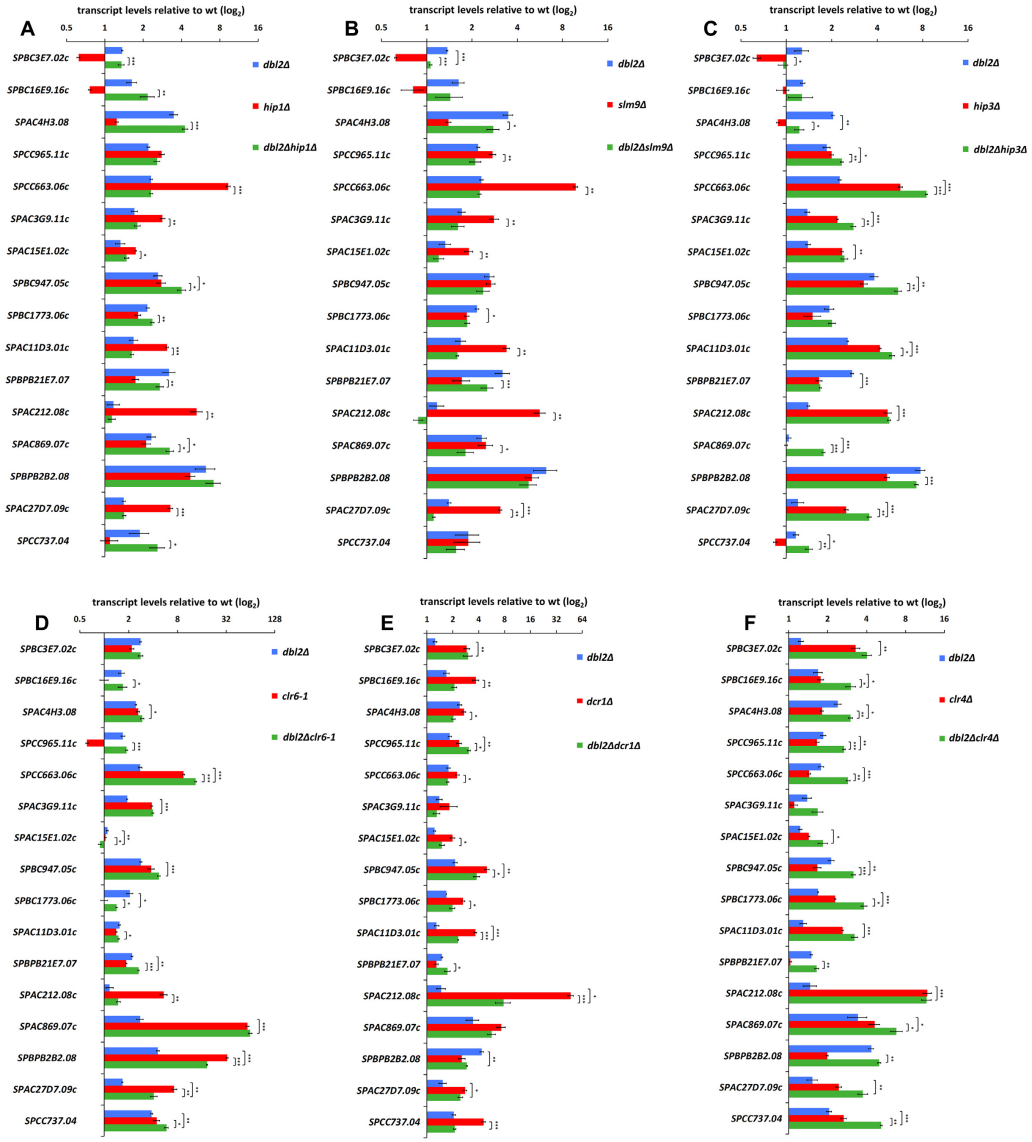
Gene expression in the *dbl2Δ* single and double mutants in combination with mutations of genes involved in gene expression. RNA was isolated from cells in the exponential phase (OD_595_ = 0.5–0.55), and gene expression was analysed using qPCR. The data represent transcript levels relative to wild-type after normalisation to *act1* and *tbp1*. The plotted values are the mean of three independent biological replicates ± standard error of the mean; asterisks denote *P* < 0.05 (*), *P* < 0.01 (**) and *P* < 0.001 (***) from two-tailed Student's *t*-tests, which was used to assess the significance of difference between the single mutants and the double mutant. (**A**) Gene expression in the *dbl2Δ* (SP067*)*, *hip1Δ* (SP456) and *dbl2Δhip1Δ* (SP467) mutants compared with wt (SP065). (**B**) Gene expression in the *dbl2Δ* (SP067*)*, *slm9Δ* (SP462) and *dbl2Δslm9Δ* (SP471) mutants compared with wt (SP065). (**C**) Gene expression in the *dbl2Δ* (SP067*)*, *hip3Δ* (SP458) and *dbl2Δhip3Δ* (SP468) mutants compared with wt (SP065). (**D**) Gene expression in the *dbl2Δ* (SP067*)*, *clr6–1* (SP415) and *dbl2Δclr6–1* (SP829) mutants compared with wt (SP065). (**E**) Gene expression in the *dbl2Δ* (SP067*)*, *dcr1Δ* (SP435) and *dbl2Δdcr1Δ* (SP501) mutants compared with wt (SP065). (**F**) Gene expression in the *dbl2Δ* (SP067*)*, *clr4Δ* (SP434) and *dbl2Δclr4Δ* (SP503) mutants compared with wt (SP065).

Significant overlap between genes upregulated in the *hip1Δ*, *slm9Δ*, *clr6–1* and *dbl2Δ* mutants prompted us to analyse the genetic interactions between the HIRA and Clr6 complexes and the *dbl2* gene. For comparison, we also included a mutant in the *clr4* methyltransferase (105), required for transcriptional silencing, and a mutant in the *dcr1* gene involved in RNAi (86,87). For epistasis analyses, we first selected several genes that were upregulated in both mutants—*dbl2Δ* and another mutant involved in the analysis. Then, we homogenized the set of genes used in these experiments. We assayed the effects of single and double mutants on the transcript levels from several loci using qPCR. Introduction of the *dbl2Δ* allele into the *hip1Δ* and *slm9Δ* background did not result in an additive increase in transcript levels compared with the *dbl2Δ* single mutant, except for *SPBC947.05c* and *SPAC869.07c* in *dbl2Δhip1Δ* (Figure 4A, B). Otherwise, the level of transcripts in the double mutants resembled that of the *dbl2Δ* single mutant; except for *SPBC3E7.02c, SPBC1773.06c* and *SPAC27D7.09c* whose transcript levels were reduced in *dbl2Δslm9Δ*. It was surprising that combining the mutation in *hip3Δ* with *dbl2Δ* led to a slight additive increase in transcript levels from half of the selected loci (*SPCC965.11c*, *SPCC663.06c*, *SPAC3G9.11c*, *SPBC947.05c*, *SPAC11D3.01c*, *SPAC869.07c*, *SPAC27D7.09c* and *SPCC737.04*) compared with any of the single mutants (Figure 4C). In addition, at three loci (*SPAC15E1.02c, SPBPB21E7.07* and *SPAC212.08c*) the double mutant showed the transcript levels similar to *hip3Δ* and at two loci (*SPBC3E7.02c* and *SPBPB2B2.08*) similar to *dbl2Δ*. Hip3 is another subunit of the HIRA complex, which consists of four subunits (Hip1, Slm9, Hip3 and Hip4). Our data suggest that Hip3 may have a cellular function that is independent of other subunits of the HIRA complex. This is also supported by the fact that genes encoding the HIRA complex exhibit different genetic interactions in *S. pombe* (48,95,101,106–108). When *clr6–1* was combined with *dbl2Δ*, the double mutant showed additive increase in the transcript levels at three measured loci (*SPCC663.06c, SPBPB21E7.07* and *SPCC737.04*) compared with any of the single mutants (Figure 4D). Moreover, at four loci (*SPBC16E9.16c*, *SPCC965.11c*, *SPAC11D3.01c* and *SPAC212.08c*), the transcript levels in *dbl2Δclr6–1* resembled that of *dbl2Δ* and equally at four loci (*SPAC4H3.08*, *SPAC3G9.11c*, *SPBC947.05c* and *SPAC869.07c*) resembled that of *clr6–1*. Comparing expression of the *dbl2Δ* and *dcr1Δ* mutants, the transcript levels in the double mutant were at seven loci (*SPBC16E9.16c*, *SPAC4H3.08*, *SPCC663.06c*, *SPAC15E1.02c*, *SPBC1773.06c*, *SPBPB21E7.07* and *SPCC737.04*) similar to *dbl2Δ* and at three loci (*SPBC3E7.02c*, *SPBPB2B2.08* and *SPAC27D7.09c*) similar to *dcr1Δ*. In addition, at one locus (*SPCC965.11c*) the level of transcripts was increased compared with any of the single mutants (Figure 4E). Finally, combining the *clr4Δ* and *dbl2Δ* mutations resulted in an additive effect on transcript levels from half of the tested loci (*SPBC16E9.16c*, *SPAC4H3.08*, *SPCC965.11c*, *SPCC663.06c*, *SPBC947.05c*, *SPBC1773.06c* and *SPCC737.04*) compared with any of the single mutants (Figure 4F). In addition, at five loci (*SPBC3E7.02c*, *SPAC15E1.02c*, *SPAC11D3.01c*, *SPAC212.08c* and *SPAC27D7.09c*) the double mutant showed transcript levels similar to the *clr4Δ* mutant and at two loci (*SPBPB21E7.07* and *SPBPB2B2.08*) similar to the *dbl2Δ* mutant. We would like to stress that not all genes behaved the same way in each mutant background. Therefore, the complex pattern of results obtained from epistasis analyses makes it difficult to draw a general conclusion about the relationship between *dbl2* and genes involved in gene repression pathways.

**Figure 5.**
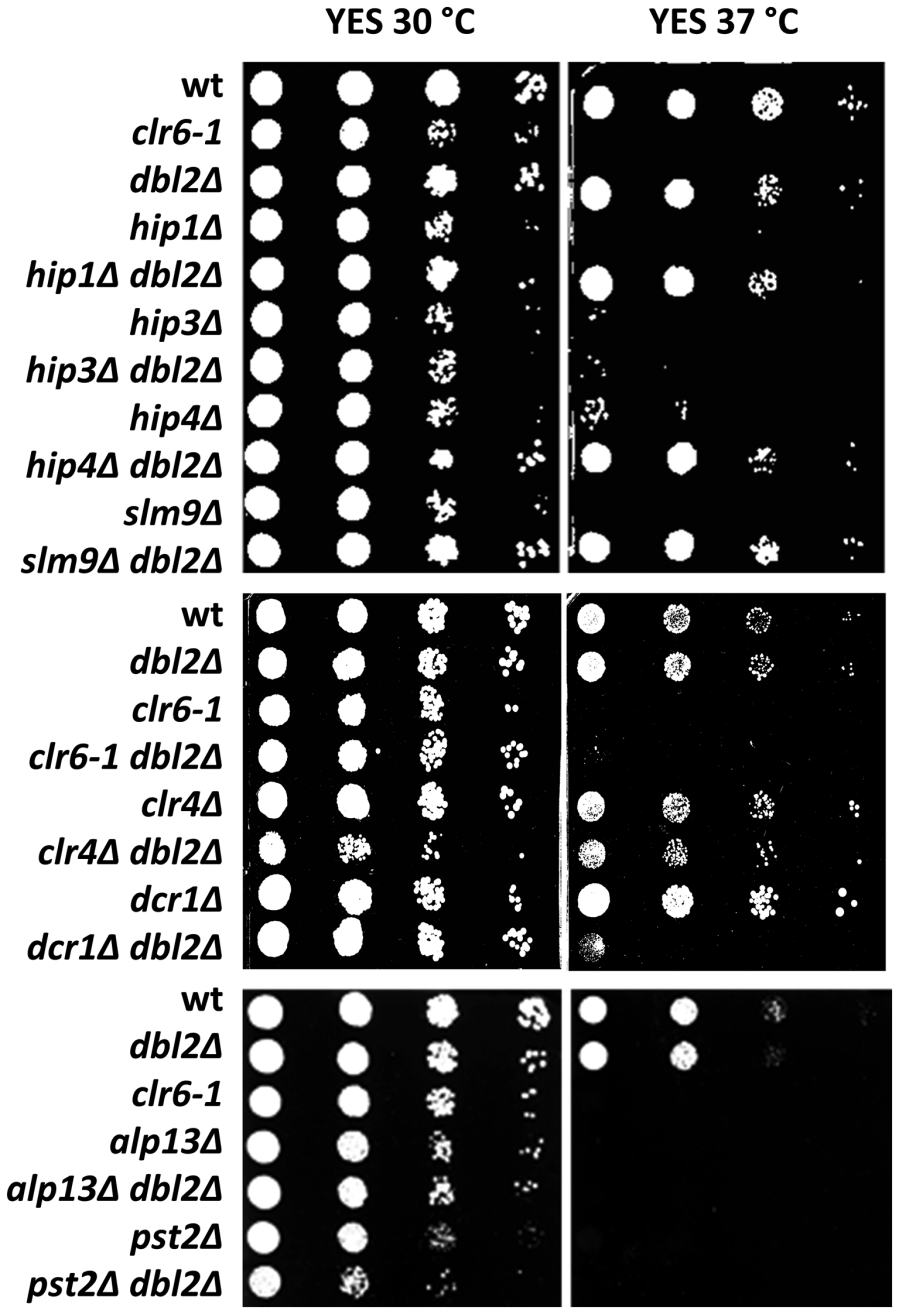
Deletion of *dbl2* suppresses the thermosensitivity of three out of four HIRA mutants, but not Clr6 complex mutants. Wild-type (SP065) and mutant strains *clr6–1* (SP415), *dbl2Δ* (SP067*)*, *hip1Δ* (SP456), *hip1Δdbl2Δ* (SP467), *hip3Δ* (SP458), *hip3Δdbl2Δ* (SP468), *hip4Δ* (SP460), *hip4Δdbl2Δ* (SP470), *slm9Δ* (SP462), *slm9Δdbl2Δ* (SP471), *clr6–1dbl2Δ* (SP829), *alp13Δ* (SP527), *dbl2Δalp13Δ* (SP553), *pst2Δ* (SP535), *dbl2Δpst2Δ* (SP561), *dcr1Δ* (SP435), *dbl2Δdcr1Δ* (SP501), *clr4Δ* (SP434) and *clr4Δdbl2Δ* (SP503) were cultivated until the exponential phase in YES medium. Tenfold serial dilutions of cell suspensions were spotted on the indicated plates. Images were taken after 3-day cultivation at 30 or 37°C (as indicated).

Given that protein–DNA interactions depend on the temperature (109), we tested the growth of single and double mutants at 37°C to further explore whether the analysed proteins function in a common or a parallel pathway. A previous study demonstrated that deletion of any subunit of the HIRA complex (*hip1Δ*, *hip3Δ*, *hip4Δ* and *slm9Δ*) results in temperature sensitivity (51). Combining mutations in *hip1Δ*, *hip4Δ* or *slm9Δ* with *dbl2Δ* rescued the growth of HIRA mutants at 37°C, suggesting that Dbl2 could act in the same pathway as the HIRA complex (Figure 5). The *dbl2Δhip3Δ* double mutant did not grow at 37°C, confirming that Hip3 behaves differently from the other members of the HIRA complex. The Clr6 complex is present in *S. pombe* in four distinct complexes (complex I, I’, I’’ and II) that share a common catalytic subunit, Clr6 (93). *Clr6-1* and the *pst2Δ* and *alp13Δ* mutants (subunits of complex II) were sensitive to higher temperature. Combining mutations in *clr6-1*, *pst2Δ* and *alp13Δ* with *dbl2Δ* did not improve growth at 37°C (Figure 5). Moreover, introduction of the *dbl2Δ* mutation into the *dcr1Δ* background resulted in temperature sensitivity, indicating some functional redundancy between these two proteins.

### Dbl2 antagonises the silencing activity of Hip1 and Slm9 at outer centromeres

**Figure 6.**
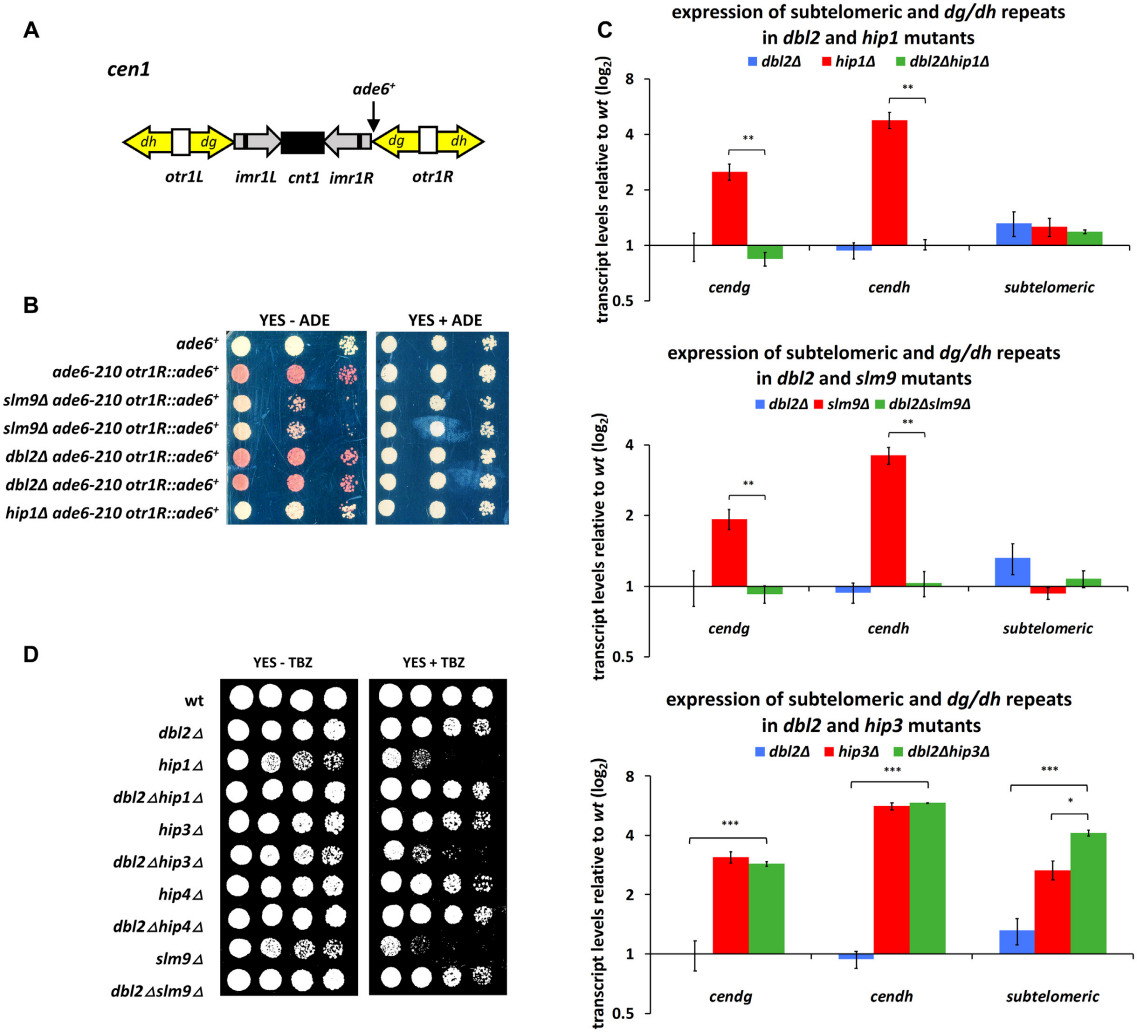
Deletion of *dbl2* suppresses the silencing defect of *hip1Δ* and *slm9Δ* mutants at the outer centromeric region. (**A**) Schematic representation of the *otr1R::ade6^+^* reporter and *dg/dh* repeats in the centromeric region. (**B**) Growth of strains on YES with limited adenine. The test strain with *ade6*^*+*^gene insert (SP392) with additional deletion of *dbl2* (SP485, SP486), *hip1* (SP489) and *slm9* (SP487, SP488) were cultivated until the exponential phase, and tenfold serial dilutions of cell suspensions were spotted on the indicated plates. Images were taken after 3-day cultivation at 30°C. (**C**) qPCR analysis of *dg/dh* repeats and subtelomeric transcripts. RNA was isolated from wild-type (SP065); single mutants *dbl2Δ* (SP067), *hip1Δ* (SP456), *slm9Δ* (SP462) and *hip3Δ* (SP458); and double mutants *dbl2Δhip1Δ* (SP467), *dbl2Δslm9Δ* (SP471) and *dbl2Δhip3Δ* (SP468) grown to the exponential phase (OD_595_ = 0.5–0.55). The data represent transcript levels relative to wild-type after normalisation to *act1* and *tbp1*. The plotted values are the mean of three independent biological replicates ± standard error of the mean; asterisks denote *P* < 0.05 (*), *P* < 0.01 (**) and *P* < 0.001 (***) from two-tailed Student's *t*-tests, which was used to assess the significance of difference between the single mutants and the double mutant. (**D**) Growth of strains on YES and YES supplemented with thiabendazole (TBZ) (15 μg/ml). The strains—wt (SP065), *dbl2Δ* (SP067), *hip1Δ* (SP456), *dbl2Δhip1Δ* (SP467), *hip3Δ* (SP458), *dbl2Δhip3Δ* (SP468), *hip4Δ* (SP460), *dbl2Δhip4Δ* (SP470), *slm9Δ* (SP462) and *dbl2Δslm9Δ* (SP471) – were cultivated until the exponential phase, and tenfold serial dilutions of cell suspensions were spotted on the indicated plates. Images were taken after 3-day cultivation at 30°C.

HIRA proteins have also been implicated in heterochromatin assembly and silencing in a range of organisms. Loss of HIRA protein in fission yeast leads to defective pericentric heterochromatin formation (101). To investigate whether Dbl2 affects pericentric heterochromatin, we tested the *dbl2Δ* mutant in a colour-based silencing assay (59). For comparison, we included deletion mutants of genes encoding HIRA subunits. As expected, HIRA null mutants alleviated silencing of the *ade6^+^*inserted at the outer centromeric repeat region (*otr1R::ade6^+^*) (Figure 6A, B). The *dbl2Δ* mutant did not show defects in the pericentric silencing (Figure 6B). We next evaluated our results by measuring expression of heterochromatic repeats at centromeres (*dg/dh*) in the single and double mutants by qPCR (Figure 6C). Consistent with our results from the colour-based assay, the *hip1Δ* and *slm9Δ* mutants displayed increased levels of pericentric transcripts, while the *dbl2Δ* mutant did not show any defect in silencing. Notably, the *dbl2Δ* mutation restored pericentric silencing in the *hip1Δ* and *slm9Δ* mutants, but not in the *hip3Δ* mutant. In addition, qPCR revealed that *dbl2, hip1* or *slm9* deletion had no effect on the expression of subtelomeric repeats (Figure 6C). We noticed an increased level of subtelomeric transcripts only in the *hip3Δ* mutant and in the corresponding double mutants with *dbl2Δ*. Furthermore, loss of pericentric heterochromatin causes impaired centromere cohesion (110,111), resulting in chromosome missegregation and sensitivity to the microtubule destabilizing drug TBZ. To test whether *dbl2* deletion in HIRA mutant cells restores pericentric heterochromatin, we analysed the sensitivity of single and double mutants to TBZ. As expected, *hip1Δ* or *slm9Δ* showed severe sensitivity to TBZ (101) (Figure 6D). Combining *dbl2Δ* with *hip1Δ* or *slm9Δ* suppressed the TBZ sensitivity of HIRA mutants (Figure 6D). In contrast, the *dbl2Δhip3Δ* double mutant showed increased sensitivity to TBZ compared with single mutants (Figure 6D), consistent with our previous qPCR results of pericentric transcripts. In conclusion, these data suggest that Dbl2 is involved in suppression of expression within the specific subnuclear domains, but in contrast to HIRA, its absence does not alleviate silencing at centromeres. However, *dbl2* deletion completely restored the silencing defect of *hip1Δ* and *slm9Δ* mutants at the outer centromere, indicating that Dbl2 may act in an antagonistic manner to Hip1 and Slm9. Notably, this phenomenon does not apply to *hip3Δ*.

### Dbl2 affects nucleosome occupancy and histone modifications

**Figure 7.**
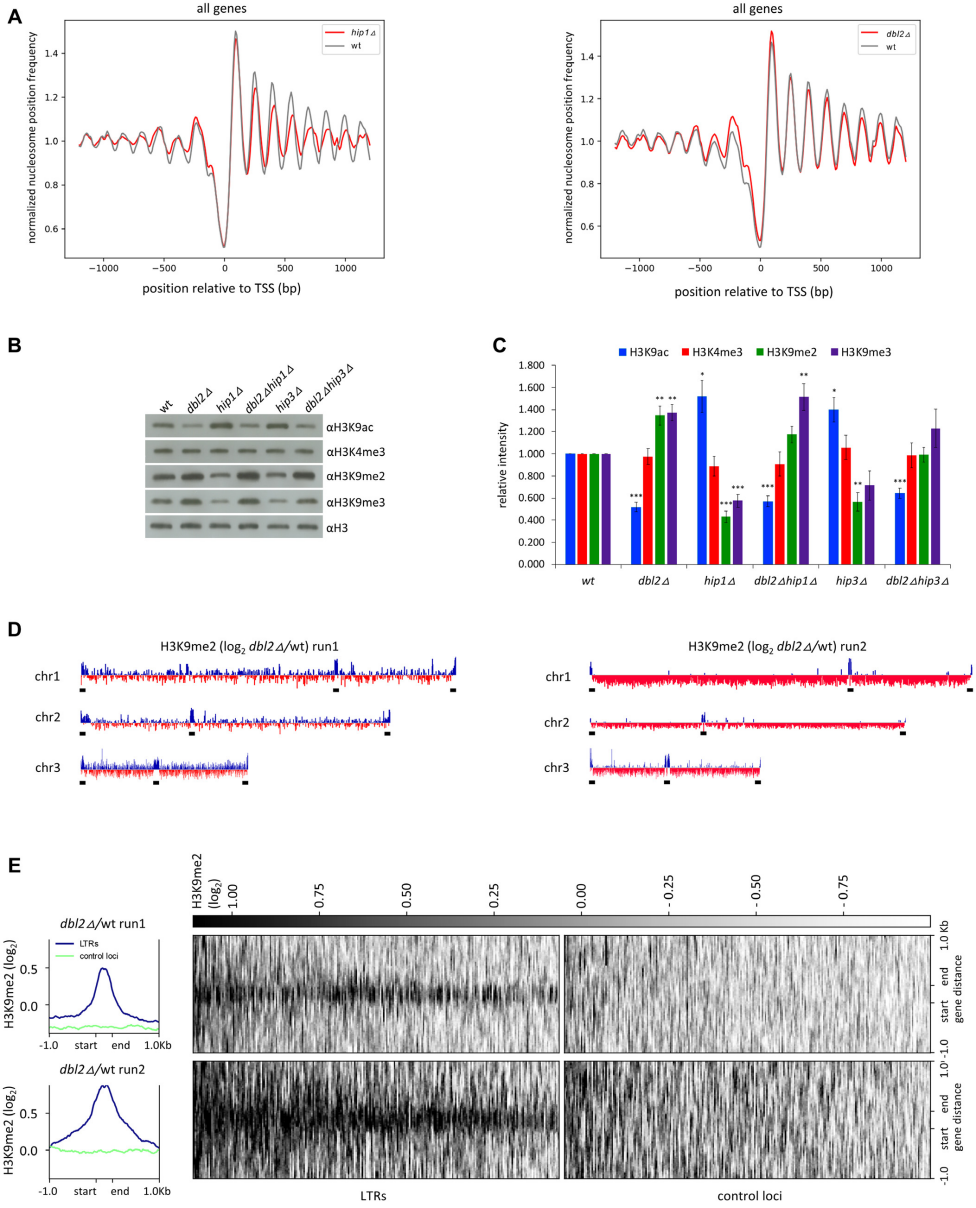
Nucleosome occupancy and histone modifications are altered in the *dbl2* and *hip1* mutants compared with the wild-type. (**A**) Average nucleosome occupancy profiles for all genes aligned at the transcription start site (TSS) in wild-type (SP072, SP065), *hip1Δ* (SP456) and *dbl2Δ* (SP067) strains. The plotted values are the mean of three independent biological replicates. To compare empirical distribution between wild-type and *dbl2Δ*, we used the Kolmogorov–Smirnov test from open-source *scipy* library, which showed statistically significant differences (*P* < 1.35 × 10^−4^). (**B, C**) Western blots were used to measure histone modifications in the indicated strains. The levels of histone modifications were normalised to total histone H3. The plotted values are the mean of four independent biological replicates ± standard error of the mean; asterisks denote *P* < 0.05 (*), *P* < 0.01 (**), and *P* < 0.001 (***) from two-tailed Student's *t*-tests, which was used to assess the significance of difference between wild-type and the mutants. (**D**) H3K9me2 occupancy at subtelomeres, centromeres and rDNA (marked by black horizontal bars) is increased in the *dbl2Δ* mutant. H3K9me2 ChIP-seq coverage in each sample was first normalised to the corresponding total H3 ChIP-seq coverage, and the *dbl2Δ* values were then further normalised to the WT values from the corresponding biological replicate. Final normalised H3K9me2 occupancy was plotted along all three fission yeast chromosomes. Results from two independent experiments are shown. The subtelomeres are here defined as regions of H3K9me2 enrichment in wild-type cells. (**E**) H3K9me2 occupancy at LTRs is increased in the *dbl2Δ* mutant. H3K9me2 occupancy normalised as in (D) was plotted for 239 LTR regions (LTR and 1 kb flanking regions) and for matched random control loci as a heatmap (right panel) or average locus profiles (left panel). Results from two independent experiments are shown.

Loss of HIRA (*hip1Δ*) leads to a global reduction in nucleosome occupancy, which is most pronounced towards the 3′ end of the genes (74). Reduced levels of specific nucleosome peaks had been detected in *hip1Δ* over individual ORFs, at Tf2 LTR retrotransposons, at some promoters and at heterochromatic repeats (74). Since our data suggest that Dbl2 and HIRA could act in the same pathway, we wondered whether Dbl2 promotes nucleosome occupancy at similar loci. We addressed this possibility by using a chromatin sequencing technology to measure genome-wide nucleosome occupancy (74,112). Chromatin derived from three biological replicates was digested with MNase to generate a DNA ladder with a highly similar molecular weight distribution between wild-type and a deletion mutant (Supplementary Figure S6A). To validate our results, we compared nucleosome patterns of wild-type and *hip1Δ* with already published results (74). Chromatin at the 5′ end of eukaryotic gene typically consists of a nucleosome-depleted region (NDR) located immediately upstream of the RNA pol II transcription start site (TSS), which is surrounded by an ordered nucleosomal array that extends into the coding sequence (113,114). As previously described, a comparison of average nucleosome positions surrounding the TSS in the *hip1Δ* mutant did not alter the NDR or the +1 nucleosome peak, but it showed reduced amplitudes of the nucleosome peaks in coding region (Figure 7A). By contrast, comparison of average nucleosome occupancy from *dbl2Δ* and wild-type revealed slightly increased amplitudes of the –2, –1 and +1 nucleosome peaks (relative to NDR) (*P* < 1.35 × 10^−4^), suggesting that Dbl2 loss could lead to increased nucleosome stability in these regions (Figure 7A). Furthermore, plots of average nucleosome profiles of Tf2 LTR retrotransposons from *hip1Δ* showed reduced nucleosome peaks downstream of the TSS and marked changes to the nucleosome positioning, mainly at the 3′-end of the genes (Supplementary Figure S6B). Consistent with our previous results, the *dbl2Δ* mutant showed slightly increased nucleosome peaks around the TSS and did not exhibit any changes to nucleosome positioning (Supplementary Figure S6B). We further confirmed these results by investigating the nucleosome profiles of both mutants at the individual genes (Supplementary Figure S7). Finally, we compared the chromatin organisation of HIRA-repressed genes (48) and Dbl2-repressed genes with the complete set of *S. pombe* coding genes. In both cases, we found that these genes exhibited lower peaks in the coding sequences and narrower and shallower NDR (Supplementary Figure S7H). This type of nucleosome profiles correlates with genes exhibiting lower median expression levels (114). Taken together, we found that Dbl2 and HIRA suppress expression of genes with similar chromatin organisation, however, unlike the *hip1Δ* mutant, the *dbl2Δ* mutant did not show any reduction in the nucleosome occupancy. On the contrary, our data suggest that in the *dbl2Δ* mutant the –2, –1 and +1 nucleosome peaks are slightly increased, indicating its role in destabilisation of the nucleosomes around the TSS.

In addition to nucleosome positioning, gene expression can be regulated through changes in histone modification patterns. In general, euchromatin is rich in hyperacetylated histones (e.g. H3K9ac) and methylated histones H3 at lysine 4 (e.g. H3K4me3), whereas heterochromatin is characterised by methylation of histone H3 at lysine 9 (e.g. H3K9me2 and H3K9me3) (115). Previous studies have shown that *hip1Δ* exhibits a substantial increase in H3K9ac levels (95). To gain further insight into Dbl2 functions, we tested the levels of histone modifications in the *dbl2Δ, hip1Δ* and *hip3Δ* single mutants and in the *dbl2Δhip1Δ* and *dbl2Δhip3Δ* double mutants using western blotting (Figure 7B, C). Our analysis showed the increased levels of H3K9ac and decreased levels of H3K9me2 and H3K9me3 in the *hip1Δ* and *hip3Δ* mutants. On the contrary, the levels of H3K9ac were significantly decreased in the single and double mutants of *dbl2Δ* and the levels of H3K9me2 and H3K9me3 were significantly increased in the *dbl2Δ* mutant. These data are in agreement with the previous conclusion that HIRA is required for histone deacetylation via Clr6 (95). Of note, with respect to histone modification, *dbl2* is epistatic to both subunits of the HIRA complex (*hip1* and *hip3*). To analyse H3K9me2 further, we performed ChIP-seq analysis (Figure 7D, E). The *dbl2Δ* mutant showed elevated levels of H3K9me2 at constitutive heterochromatic regions, i.e., centromeres, subtelomeres and rDNA compared with the wild-type cells (Figure 7D, Supplementary Figure S8). Moreover, a closer examination of the data revealed elevated levels of H3K9me2 at LTRs in the *dbl2Δ* mutant (Figure 7E). Taken together, these results suggest that although gene expression in the *dbl2Δ* mutant is mostly increased, Dbl2 is required for nucleosome destabilisation and reduction of H3K9me2 at centromeres, subtelomeres, rDNA regions and LTRs.

### Proteins involved in HR repress expression from the same genomic regions as Dbl2

**Figure 8.**
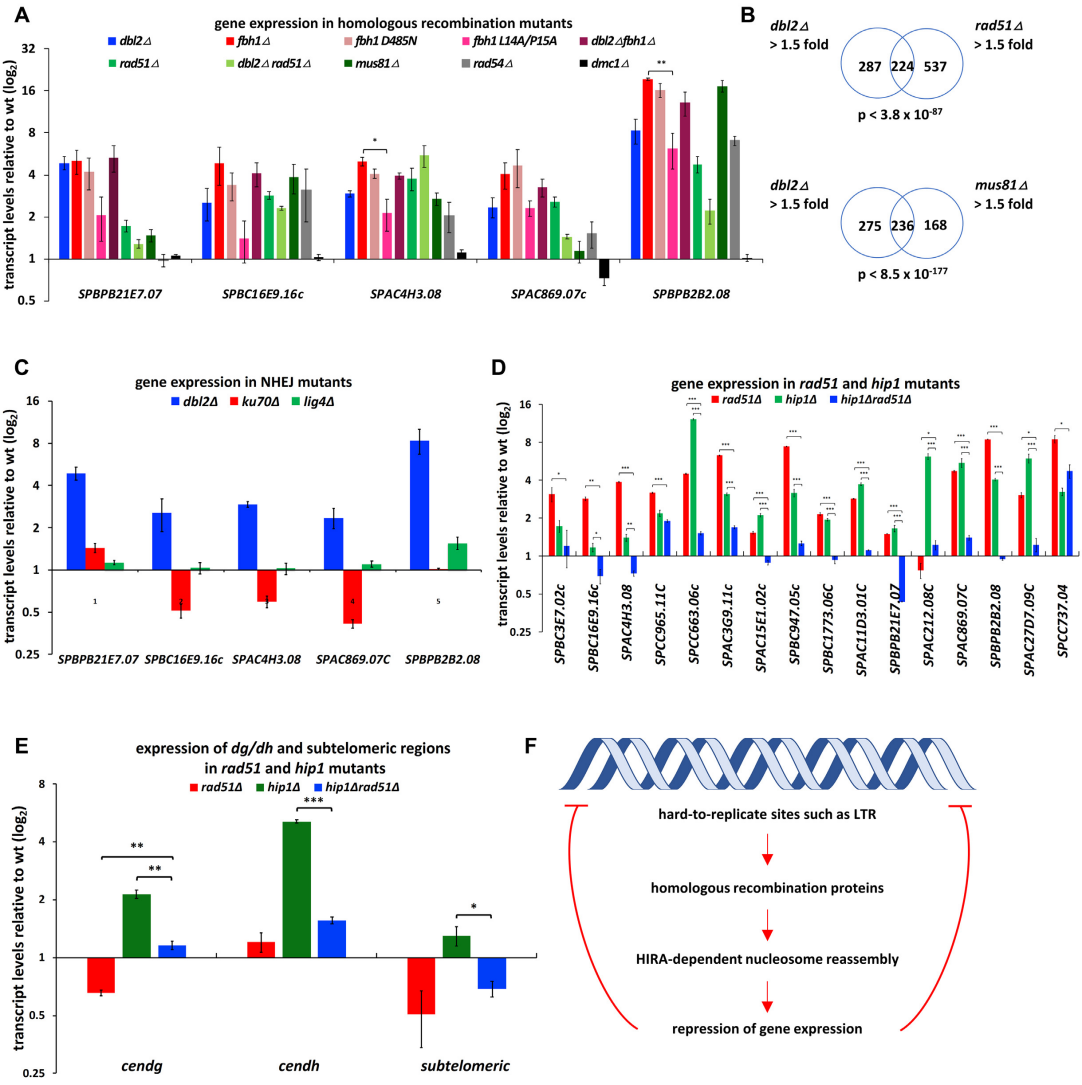
Cells lacking homologous recombination proteins show expression profiles similar to *dbl2Δ*. (**A**) RNA was isolated from wild-type (SP065), *dbl2Δ* (SP067), *fbh1Δ* (SP070), *fbh1*^D485N^ (SP633), *fbh1*^L14A/P15A^ (SP629), *dbl2Δfbh1Δ* (SP613), *rad51Δ* (SP068), *dbl2Δrad51Δ* (SP735), *mus81Δ* (SP375), *rad54Δ* (SP025) and *dmc1Δ* (SP069) strains in the exponential phase, and gene expression was analysed using qPCR. The data represent transcript levels relative to wild-type after normalisation to *act1* and *tbp1*. The plotted values are the mean of three independent biological replicates ± standard error of the mean; asterisks denote *P* < 0.05 (*), *P* < 0.01 (**) and *P* < 0.001 (***) from two-tailed Student's *t*-tests, which was used to assess the significance of difference between *fbh1Δ* and *fbh1*^L14A/P15A^. (**B**) Venn diagrams showing overlap between genes upregulated in the *dbl2Δ* mutant (SP067) and genes upregulated in the *rad51Δ* (SP068) or *mus81Δ* (SP375) mutant. Only genes included in both analyses were compared. The *P*-value indicates the probability that the observed overlap happened by chance. (**C**) RNA was isolated from wild-type (SP065), *dbl2Δ* (SP067), *ku70Δ* (SP700) and *lig4Δ* (SP842) strains in exponential phase and gene expression was analysed using qPCR. The *dbl2Δ* strain was used as a positive control. (**D, E**) RNA was isolated from wild-type (SP065), *rad51Δ* (SP068), *hip1Δ* (SP456) and *rad51Δhip1Δ* (SP793) strains in the exponential phase and gene expression was analysed using qPCR. The plotted values are the mean of three independent biological replicates ± standard error of the mean; asterisks denote *P* < 0.05 (*), *P* < 0.01 (**) and *P* < 0.001 (***) from two-tailed Student's *t*-tests, which was used to assess the significance of difference between the single mutants and the double mutant. (**F**) Model of repression of gene expression near hard-to-replicate sites such as repetitive sequences. In wild-type cells, hard-to-replicate sites trigger replication fork stalling or collapse, which is associated with the generation of single-stranded or double-stranded DNA breaks. Homologous recombination proteins along with the HIRA histone chaperone ensure that repressive chromatin is assembled and genome integrity is maintained.

In HR, Dbl2 is required for the formation of Fbh1 DNA helicase foci in order to remove Rad51 protein and to process DNA joint molecules (15). To address whether Dbl2 also exerts its function through Fbh1 in the process of the repression of gene expression, we assayed the effects of *fbh1Δ* and *dbl2Δ* single and double mutants on transcript levels from several loci using qPCR. The transcript levels in *fbh1Δ* at measured loci were similar to those in *dbl2Δ;* combining the *dbl2Δ* and *fbh1Δ* mutations did not show any additive increase in transcript levels compared with the single mutants (Figure 8A). To further examine this phenomenon, we assayed the transcript levels in the F-box (*fbh1*^L14A/P15A^) *fbh1* mutant and in the helicase-defective (*fbh1*^D485N^) *fbh1* mutant (13). The transcript levels in the *fbh1*^D485N^ mutant were comparable to those in the *fbh1Δ* mutant, whereas the *fbh1*^L14A/P15A^ mutant also showed increased – albeit to a lesser extent – transcript levels, indicating that Fbh1 and mainly its helicase domain function with Dbl2 in the repression of gene expression.

A key Fbh1 function is to disrupt Rad51 nucleoprotein filaments via two activities: DNA unwinding/translocation and ubiquitin ligation (12). We therefore hypothesised that in the *dbl2Δ* or *fbh1Δ* mutants the level of transcripts at measured loci will be affected by Rad51 accumulation. We tested this hypothesis by analysing a *rad51Δ* deletion mutant (15). To our surprise, the *rad51Δ* mutant showed increased transcript levels similar to the *dbl2Δ* mutant (except for *SPBPB21E7.07*), suggesting that the presence of Rad51 protein is necessary to repress gene expression. The corresponding double mutant with *dbl2Δ* displayed decreased transcript levels of four tested loci and a slight increase (statistically not significant) of *SPAC4H3.08* compared with the single mutants (Figure 8A). These unexpected results prompted us to assay the level of transcripts in deletion mutants of other genes encoding further proteins involved in HR. We selected Rad54, which enables D-loop formation (22–24) and promotes DNA strand invasion by the Rad51 presynaptic filament (19–21), and Mus81, which is a subunit of the Mus81–Eme1 Holliday junction resolvase (14,31,116–119). Both *rad54Δ* and *mus81Δ* deletion mutants showed increased transcript levels at measured loci. In order to validate the qPCR data, we compared the expression profiles of the *rad51Δ* and *mus81Δ* mutants with those of wild-type by using strand-specific RNA-seq (Figure 8B, Supplementary Table S3). As expected, the genes that were upregulated in *dbl2Δ* were highly correlated with upregulated genes in the *rad51Δ* and *mus81Δ* mutants (*P* < 3.8 × 10^−87^ and *P* < 8.5 × 10^−177^, respectively). To exclude the possibility that the altered expression profiles are due to the presence of the deletion cassettes used to generate corresponding mutants, we analysed the impact of the *dmc1* gene deletion. Dmc1 is a meiosis-specific recombinase that mediates homologous DNA pairing (120), so its deletion in mitotically dividing cells should not cause any effect. Using qPCR, we detected no change in expression of measured genes in the *dmc1Δ* strain. We next asked whether genes involved in NHEJ are also required for gene repression. Using qPCR, we assayed transcript levels in deletion mutants of genes encoding protein Pku70 and the DNA repair ligase Lig4 (Figure 8C). The Ku70/Ku80 complex binds dsDNA ends, inhibits end-resection and allows the ligation of DSBs by Lig4 (1,121,122). Cells lacking Pku70 or Lig4 did not show any increase in transcript levels (Figure 8C), suggesting that the NHEJ pathway is not required for gene repression at measured loci.

The ability of *dbl2Δ* to suppress the silencing defect of *hip1Δ* and *slm9Δ* mutants at pericentric region prompted us to test genetic interactions between *rad51* and *hip1*. The *rad51Δ* and *hip1Δ* double mutants showed mostly decreased transcript levels compared with the single mutants at genomic loci (Figure 8D). Although the nature of the decreased transcript levels in the double mutants remains unclear, the non-additive phenotype of the double mutants suggests that they could act in the same pathway. Furthermore, deletion of *rad51Δ* restored the silencing defect of *hip1Δ* at the outer centromere region (Figure 8E). In addition, combining mutations in *hip1Δ* or *slm9Δ* with *rad51Δ* partially rescued the growth of all mutants at 37°C (Supplementary Figure S9). In conclusion, these results suggest a coordinated pathway involving proteins of HR and the HIRA complex in the repression of a large number of genes. It is possible that this coordinated action of HR and HIRA also contributes to the regulation of centromeric silencing.

## DISCUSSION

We present here that the Dbl2 protein along with other proteins involved in HR or replication fork integrity, such as Fbh1, Rad51, Rad54 and Mus81, are required for repression of gene expression (Figure 8). We linked the Dbl2 protein to the HIRA histone chaperone by genetic interactions (Figures 4–7). Our data suggest a possible role for HR proteins in coordinating nucleosome occupancy to assemble repressive chromatin at a large number of genes, which are close to repeat sequences.

Our results suggest that Dbl2 could function in the same pathway as the HIRA complex regarding repression of gene expression at euchromatic and heterochromatic loci. Notably, cells lacking either Dbl2 or HIRA factors (Hip1 or Slm9) exhibit increased levels of antisense RNA and RNA from the Tf2 LTR retrotransposons, subtelomeric genes and meiotic genes (48,51,95). The transcript levels in the *hip1Δdbl2Δ* or *slm9Δdbl2Δ* double mutants are mostly similar to that of *dbl2Δ* single mutant, indicating that Dbl2 might act upstream of HIRA (Figures 4A, B and 6C). Moreover, introduction of the *dbl2Δ* mutation into the *hip1Δ, hip4Δ* and *slm9Δ* background rescues the growth of HIRA mutants at 37°C (Figure 5) or in the presence of TBZ (Figure 6D). Furthermore, genetic interactions between Dbl2 and HIRA extend to histone modifications, because introduction of *dbl2Δ* into *hip1Δ* and *hip3Δ* reduces H3K9ac to levels found in the *dbl2Δ* mutant (Figure 7B, C).

There are two main pathways in *S. pombe* that repress antisense expression at euchromatic loci. The Clr4 complex and RNAi factors (e.g. Dcr1) mediate degradation of read-through antisense RNA via a mechanism involving nuclear exosome (88), and a pathway involving the Clr6, Set2 and the HIRA complex represses initiation of antisense transcripts from cryptic promoters (93,95). At some loci *dbl2* acts in a redundant manner with *clr4* (Figure 4F) and genes with increased antisense levels in the *dbl2Δ* mutant do not map preferentially to convergent loci (49%). These findings suggest that Dbl2 rather acts in a pathway involving HIRA. However, the epistasis analyses regarding repression of gene expression at euchromatic loci showed a very complex pattern of interactions between *dbl2* and *clr4*, *dcr1, clr6-1* and *hip3* (Figure 4). Therefore, these results cannot be used to unambiguously describe genetic pathways involved in these processes.

A previous report revealed that cells lacking the HIRA histone chaperone experience a global reduction in nucleosome occupancy at both euchromatic and heterochromatic loci (74). Our results indicate that nucleosome peaks around the TSS are slightly increased in the *dbl2Δ* mutant compared with the wild-type (Figure 7A), indicating that Dbl2 could be involved in removing excess nucleosomes. In support of this notion, Rad51 overexpression stimulates the histone eviction at DSBs in *bre1Δ* mutant (123). In wild-type cells, Bre1 stimulates histone eviction at DSBs by H2B ubiquitination (123). Furthermore, a recent study analysing regulators of heterochromatin spreading in different chromatin contexts demonstrated that the HIRA histone chaperone and genes involved in HR pathway, such as *rad50* and *sfr1*, act in opposite ways (124). The authors detected these actions mainly at the ectopic heterochromatin domain composed of *dh* element embedded in gene-rich euchromatin. The HIRA complex has been implicated in heterochromatic domains expansion, while *rad50* and *sfr1* have been found to function in heterochromatin destabilisation (124). Consistently, we show that H3K9me2 nucleosomes are elevated in the *dbl2Δ* mutant at constitutive heterochromatic regions and LTRs (Figure 7D, E). It remains unclear how these chromatin changes could increase gene expression in the *dbl2Δ* mutant, although it has been shown that H3K9me2 domains are transcriptionally active and contain modifications associated with euchromatic transcription (125). Additional studies in HR-deficient mutants should be now conducted in order to establish how HR proteins affect chromatin status and stability.

Both the Dbl2 protein and the HIRA complex are involved in repression of Tf2 LTR retrotransposons. Tf2 LTR retrotransposons are organised into Tf bodies, the function of which depends on CENP-B (Abp1, Cbh1 and Cbh2), the Set1 histone methyltransferase (126) and HDAC proteins (Clr6, Clr3, and Hst4) (92,127–129). The RNAi machinery plays only an accessory role to the exosome in this process (82,130,131). Importantly, CENP-B-mediated inhibition of Tf2 LTR transcription is independent of the repressive function of HIRA complex (128). The positive genetic interactions between *dbl2* and HIRA suggest that HIRA and Dbl2 might represent a common pathway of Tf2 repression distinct from CENP-B regulation (132). However, it still needs to be experimentally confirmed. In human cells after infection with naked viral DNAs, HIRA co-localises with viral genomes, binds to incoming viral DNAs and deposits H3.3 onto them (133). Given the parallels between Tf2 and viral DNA, it will be interesting to determine whether the human ortholog of Dbl2 (ZGRF1) is also required for repression of viral DNA.

It is surprising that *hip3* behaves differently from the other members of the HIRA complex (Figures 4, 5 and 6). The *dbl2Δhip3Δ* double mutant showed at some loci a slight additive increase in transcript levels compared with the single mutants (Figures 4C and 6C), and introduction of *dbl2Δ* did not rescue the growth of *hip3Δ* at 37°C or on TBZ (Figures 5 and 6D). These findings indicate a very different, but currently unknown, function for Hip3 in gene expression. Nevertheless, these results are consistent with a previous report that linked different physical interactions, involving HIRA (ortholog of Hip1 and Slm9), UBN1 (ortholog of Hip4) and ASF1, to the distinct functional properties of active chaperone complexes in HeLa cells (134). Systematic mapping of Hip3 physical and genetic interactions will be required to better understand its function in gene expression and chromatin remodelling.

Our analyses further revealed that other proteins involved in HR, such as Fbh1, Rad51, Rad54 and Mus81, are also required for the repression of gene expression (Figure 8A, B). The role of HR proteins in the regulation of gene expression has also been observed in other organisms. In human cell lines with deficiency in HR repair genes, expression of numerous genes required for DNA damage repair, cell cycle regulation and DNA replication is upregulated or downregulated (135). For example, the expression levels of three genes encoding DSB end resection enzymes, *BLM*, *DNA2* and *EXO1*, are all reduced in HR-deficient cell lines created by depletion of independent HR genes (135). In plants, HR proteins such as RAD51D, RAD51 and XRCC2 have been shown to regulate expression of pathogen-related genes and genes important for DNA repair, abiotic stress and transcriptional regulation (136–138). Our RNA-seq analysis of the *dbl2Δ* mutant did not reveal differentially expressed protein-coding genes involved in DNA repair, but there were differentially expressed genes involved in meiosis, detoxication and transport. Moreover, these misregulated loci in the *dbl2Δ* mutant are near repetitive DNA elements, such as long tandem and long terminal repeats (Figure 2). However, this correlation between the localisation of misregulated genes and the localisation of LTRs may be an indirect consequence of the preference of Tf LTR retrotransposons to insert in the promoters of certain types of genes (139,140). Our findings are also similar to recent studies that have implicated HR proteins in the repression of repeat sequences and transposable elements. Loss of Brca1 in mice results in transcriptional derepression of satellite DNA sequences (141). Furthermore, the loss of RAD51 and ATR checkpoint kinase triggers satellite expression and the loss of SET-25 methyltransferase leads to expression of retrotransposons and tissue-specific genes in *Caenorhabditis elegans* (142).

### How do HR and HIRA orchestrate the assembly of repressive chromatin?

Our data provide evidence that cells lacking Dbl2 exhibit increased levels of transcripts located closer to long terminal repeats and long tandem repeats compared with the loci with no change in expression. LTRs in *S. pombe* impair DNA replication by blocking fork progression, a property that results from Sap1 binding to LTRs (132). The authors proposed that the function of this block is to control the direction of transposon replication, possibly by coordinating lagging strand synthesis, which prevents single strand annealing from complementary direct repeats (132). CENP-B factors counteract Sap1 activity and promote replication fork progression through the LTRs (132). Blocked replication forks are potential sources of genome instability because they can lead to replisome collapse and DSB formation (143). Consistent with the fact that LTR regions are prone to replication fork stalling, some of the loci misregulated in *dbl2Δ* overlap with genomic loci covered with γH2A (Figure 2C) (85). Transient formation of γH2A is assumed to result from replication fork arrest or collapse during the S-phase (85). If the LTR loci in question are constantly experiencing stalled replication forks during normal replication, their DNA damage and repair load should be higher, which should result in a different histone landscape compared with other chromosomal regions, which keeps replenishing itself by continuous cell division. This finding is in agreement with our results that the *dbl2Δ* cells grown in the stationary phase did not show significant changes in gene expression (Supplementary Figure S2). We propose that Dbl2 and other HR proteins are implicated in repression of genes located near hard-to-replicate sites such as LTRs (Figure 8B). The involvement of Mus81–Eme1 and Rad54 in this process suggests that stalled replication forks could be converted into DSBs (144–148) and processed by HR. In the absence of HR proteins, other pathways such as NHEJ should be involved in DSB repair. A study recently reported that several chromatin modifications which occur proximal to DSBs, are indeed pathway-specific (149). Additional studies should now be conducted to establish whether different histone modifications depending on HR or NHEJ repair are established at these regions and how they affect gene expression.

It is notable that in minimal medium we observed much smaller changes in gene expression in the *dbl2Δ* mutant (Supplementary Figure S2). The specific effect of Dbl2 under YES growth conditions is not clear, however, the data may reflect either more replication–transcription collisions under rapid growth conditions or the environmental control of the epigenetic state. Recent experiments determining the role of TOR2, a major regulator of eukaryotic cellular growth, revealed that epigenetic stability of subtelomeric chromatin responds to cellular growth conditions (150). This is remarkably similar to significant upregulation of genes at the subtelomeric regions of chromosomes I and II in *dbl2Δ* (chi-squared test, *P* < 2.12 × 10^−49^). Whether the effect of Dbl2 and TOR2 is related remains to be investigated.

Our data suggest a link between Dbl2 and the HIRA histone chaperone. A recent study showed that *S. pombe* cells lacking HIRA experience a global reduction in nucleosome occupancy at gene sequences, although there are some regions of the genome that are more severely perturbed (74). Our observation that HR and HIRA could act in the same pathway would explain why specific regions of the genome show greater dependency upon HIRA than others. Replication protein A (RPA), which is best known for its role in DNA repair and replication (151), has been recently implicated in human cells also in the efficient deposition of newly synthesised H3.3 by the HIRA histone chaperone at promoters and enhancers (152). Although it is presently unclear how this process is elicited at a mechanistic level, it is tempting to speculate that Dbl2 in concert with HR may contribute to the eviction of histones followed by HIRA-mediated nucleosome occupancy to assemble repressive chromatin near long terminal and long tandem repeats (Figure 8F).

## DATA AVAILABILITY

RNA-seq data are available at the National Center for Biotechnology Information (NCBI) under accession numbers PRJNA601919 and PRJNA659385. The ChIP-seq data are available at the ArrayExpress database (www.ebi.ac.uk/arrayexpress) under an accession number E-MTAB-9619. The scripts used for ChIP-seq data processing and analyses are available at https://github.com/mprevorovsky/bagelova-polakova-dbl2-histones.

## Supplementary Material

gkab027_Supplemental_Files
