# Supplementary material for: Repression of a large number of genes requires interplay between homologous recombination and HIRA
Source: Nucleic Acids Res. 2021 Jan 28;49(4):1914–34. doi: 10.1093/nar/gkab027 (PMC7913671; doi:10.1093/nar/gkab027)
Supplement: gkab027_Supplemental_Files [file gkab027_supplemental_files.zip › Supplementary Material 05012021.pdf]

Supplementary Material

Table S1. *S. pombe* strains

Strain ID	Genotype	Source	Original strain
**SP025**	*h$^+$ rad54::kanMX4 (omns)*[a]	J. Gregan	JG17817
**SP065**	*h$^-$ leu1-32 ura4-D18 ade6-M210*	J. Gregan	JG11318
**SP067**	*h$^-$ leu1-32 ura4-D18 ade6-M210 dbl2::natMX4*	J. Gregan	JG17207
**SP068**	*h$^+$ rad51::hphMX4 (omns)*[a]	J. Gregan	JG17540
**SP069**	*h$^+$ leu1-32 ade6-M216 ura4-D18 dmc1::kanMX4*	Bioneer	ED668h$^+$
**SP070**	*h^{90} fbh1::kanMX4 (omns)* [a]	J. Gregan	JG17544
**SP072**	*h$^+$ ade6-M210 ura4-D18 leu1-32*	Bioneer	ED666h$^+$
**SP375**	*h^{90} mus81::kanMX4 (omns)* [a]	J. Gregan	JG17884
**SP392**	*h$^+$ ade6-210 leu1-32 ura4-D18 otr1R(Sph1)::ade6*	R. Allshire	FY1180
**SP415**	*h$^+$ leu1 his2 clr6-1*	NBRP Japan	FY11920
**SP434**	*h$^+$ leu1 his2 ura4 clr4::Ura4*	NBRP Japan	FY20743
**SP435**	*h$^+$ leu1 his2 dcr1::kanMX4*	NBRP Japan	FY20473
**SP456**	*h$^+$ ade6-M210 ura4-D18 leu1-32 hip1::kanMX4*	this work	SP072
**SP458**	*h$^+$ ade6-M210 ura4-D18 leu1-32 hip3::kanMX4*	this work	SP072
**SP460**	*h$^+$ ade6-M210 ura4-D18 leu1-32 hip4::kanMX4*	this work	SP072
**SP462**	*h$^+$ ade6-M210 ura4-D18 leu1-32 slm9::kanMX4*	this work	SP072
**SP467**	*h$^-$ leu1-32 ura4-D18 ade6-M210 dbl2::natMX4 hip1::kanMX4*	this work	SP067
**SP468**	*h$^-$ leu1-32 ura4-D18 ade6-M210 dbl2::natMX4 hip3::kanMX4*	this work	SP067
**SP470**	*h$^-$ leu1-32 ura4-D18 ade6-M210 dbl2::natMX4 hip4::kanMX4*	this work	SP067
**SP471**	*h$^-$ leu1-32 ura4-D18 ade6-M210 dbl2::natMX4 slm9::kanMX4*	this work	SP067
**SP485**	*h$^+$ ade6-210 leu1-32 ura4-D18 otr1R(Sph1)::ade6 dbl2::natMX4*	this work	SP392
**SP486**	*h$^+$ ade6-210 leu1-32 ura4-D18 otr1R(Sph1)::ade6 dbl2::natMX4*	this work	SP392
**SP487**	*h$^+$ ade6-210 leu1-32 ura4-D18 otr1R(Sph1)::ade6 slm9::kanMX4*	this work	SP392
**SP488**	*h$^+$ ade6-210 leu1-32 ura4-D18 otr1R(Sph1)::ade6 slm9::kanMX4*	this work	SP392
**SP489**	*h$^+$ ade6-210 leu1-32 ura4-D18 otr1R(Sph1)::ade6 hip1::kanMX4*	this work	SP392
**SP501**	*dcr1::kanMX4 dbl2::natMX4 (omns)* [a]	this work	SP067, SP435

**SP503**	*clr4::Ura4 dbl2::natMX4 (omns)* [a]	this work	SP067, SP434
**SP527**	*h*$^{+}$ *leu1-32 ade6-M216 ura4-D18 alp13::kanMX4*	Bioneer	
**SP535**	*h*$^{+}$ *leu1-32 ade6-M216 ura4-D18 pst2::kanMX4*	Bioneer	
**SP553**	*alp13::kanMX4 dbl2::natMX4 (omns)* [a]	this work	SP067, SP527
**SP561**	*pst2::kanMX4 dbl2::natMX4 (omns)* [a]	this work	SP067, SP535
**SP613**	*h*^{90} *fbh1::kanMX4 dbl2::natMX4 (omns)* [a]	J. Gregan	JG17545
**SP629**	*h*$^{+}$ *fbh1 L14A/P15A-kanR ura4-D18 leu1-32 his3-D1 arg3-D4*	M. Whitby	MCW1768
**SP633**	*h*$^{+}$ *fbh1 D485N-kanR ura4-D18 leu1-32 his3-D1 arg3-D4*	M. Whitby	MCW1718
**SP700**	*h*$^{+}$ *pku70::kanMX6 leu1-32 ura4-D18 his2*	NBRP Japan	FY23686
**SP735**	*rad51::hphMX4 dbl2::natMX4 (omns)* [a]	J. Gregan	JG17507
**SP792**	*rad51::hphMX4 hip1::kanMX4 (omns)* [a]	this work	SP68, SP456
**SP793**	*rad51::hphMX4 hip1::kanMX4 (omns)* [a]	this work	SP68, SP456
**SP796**	*rad51::hphMX4 slm9::kanMX4 (omns)* [a]	this work	SP68, SP462
**SP797**	*rad51::hphMX4 slm9::kanMX4 (omns)* [a]	this work	SP68, SP462
**SP829**	*dbl2::natMX4 clr6-1 (omns)* [a]	this work	SP067, SP415
**SP842**	*h*$^{+}$ *lig4::kanMX4 ade6-M21? ura4-D18 leu1-32*	NBRP Japan	FY25695
**SP1045**	*h*$^{-}$ *ade6-M216 leu1-32 ura4-D18 Tf2-1::lacZ (ura4*$^{+}$*)*	S. Whitehall	JWP1
**SP1046**	*h*$^{-}$ *ade6-M216 leu1-32 ura4-D18 Tf2-2::lacZ (ura4*$^{+}$*)*	S. Whitehall	JWP2
**SP1047**	*h*$^{-}$ *ade6-M216 leu1-32 ura4-D18 Tf2-3::lacZ (ura4*$^{+}$*)*	S. Whitehall	JWP3
**SP1048**	*h*$^{-}$ *ade6-M216 leu1-32 ura4-D18 Tf2-4::lacZ(ura4*$^{+}$*)*	S. Whitehall	JWP4
**SP1049**	*h*$^{-}$ *ade6-M216 leu1-32 ura4-D18 Tf2-5::lacZ(ura4*$^{+}$*)*	S. Whitehall	JWP5
**SP1050**	*h*$^{-}$ *ade6-M216 leu1-32 ura4-D18 Tf2-6::lacZ(ura4*$^{+}$*)*	S. Whitehall	JWP6
**SP1051**	*h*$^{-}$ *ade6-M216 leu1-32 ura4-D18 Tf2-7::lacZ(ura4*$^{+}$*)*	S. Whitehall	JWP7
**SP1052**	*h*$^{-}$ *ade6-M216 leu1-32 ura4-D18 Tf2-8::lacZ(ura4*$^{+}$*)*	S. Whitehall	JWP8
**SP1053**	*h*$^{-}$ *ade6-M216 leu1-32 ura4-D18 Tf2-9::lacZ(ura4*$^{+}$*)*	S. Whitehall	JWP9
**SP1054**	*h*$^{-}$ *ade6-M216 leu1-32 ura4-D18 Tf2-10::lacZ(ura4*$^{+}$*)*	S. Whitehall	JWP10
**SP1055**	*h*$^{-}$ *ade6-M216 leu1-32 ura4-D18 Tf2-11::lacZ(ura4*$^{+}$*)*	S. Whitehall	JWP11
**SP1056**	*h*$^{-}$ *ade6-M216 leu1-32 ura4-D18 Tf2-12::lacZ(ura4*$^{+}$*)*	S. Whitehall	JWP12
**SP1057**	*h*$^{-}$ *ade6-M216 leu1-32 ura4-D18 Tf2-13::lacZ(ura4*$^{+}$*)*	S. Whitehall	JWP13
**SP1058**	*h*$^{-}$ *ade6-M216 leu1-32 ura4-D18 Tf2-1::lacZ(ura4*$^{+}$*) hip1::ura4*$^{+}$	S. Whitehall	JWP14
**SP1059**	*h*$^{-}$ *ade6-M216 leu1-32 ura4-D18 Tf2-2::lacZ(ura4*$^{+}$*) hip1::ura4*$^{+}$	S. Whitehall	JWP15
**SP1060**	*h*$^{-}$ *ade6-M216 leu1-32 ura4-D18 Tf2-3::lacZ(ura4*$^{+}$*) hip1::ura4*$^{+}$	S. Whitehall	JWP16
**SP1061**	*h*$^{-}$ *ade6-M216 leu1-32 ura4-D18 Tf2-4::lacZ(ura4*$^{+}$*) hip1::ura4*$^{+}$	S. Whitehall	JWP17
**SP1062**	*h*$^{-}$ *ade6-M216 leu1-32 ura4-D18 Tf2-5::lacZ(ura4*$^{+}$*)*	S. Whitehall	JWP18

	hip1::ura4$^+$		
**SP1063**	h^- ade6-M216 leu1-32 ura4-D18 Tf2-6::lacZ(ura4$^+$)<br>hip1::ura4$^+$	S. Whitehall	JWP19
**SP1064**	h^- ade6-M216 leu1-32 ura4-D18 Tf2-7::lacZ(ura4$^+$)<br>hip1::ura4$^+$	S. Whitehall	JWP20
**SP1065**	h^- ade6-M216 leu1-32 ura4-D18 Tf2-8::lacZ(ura4$^+$)<br>hip1::ura4$^+$	S. Whitehall	JWP21
**SP1066**	h^- ade6-M216 leu1-32 ura4-D18 Tf2-9::lacZ(ura4$^+$)<br>hip1::ura4$^+$	S. Whitehall	JWP22
**SP1067**	h^- ade6-M216 leu1-32 ura4-D18 Tf2-10::lacZ(ura4$^+$)<br>hip1::ura4$^+$	S. Whitehall	JWP23
**SP1068**	h^- ade6-M216 leu1-32 ura4-D18 Tf2-11::lacZ(ura4$^+$)<br>hip1::ura4$^+$	S. Whitehall	JWP24
**SP1069**	h^- ade6-M216 leu1-32 ura4-D18 Tf2-12::lacZ(ura4$^+$)<br>hip1::ura4$^+$	S. Whitehall	JWP25
**SP1070**	h^- ade6-M216 leu1-32 ura4-D18 Tf2-13::lacZ(ura4$^+$)<br>hip1::ura4$^+$	S. Whitehall	JWP26
**SP1075**	h^- ade6-M216 leu1-32 ura4-D18 Tf2-1::lacZ(ura4$^+$)<br>dbl2:: natMX4	this work	JWP1,<br>SP1115
**SP1076**	h^- ade6-M216 leu1-32 ura4-D18 Tf2-1::lacZ(ura4$^+$)<br>dbl2:: natMX4	this work	JWP1,<br>SP1115
**SP1077**	h^- ade6-M216 leu1-32 ura4-D18 Tf2-1::lacZ(ura4$^+$)<br>dbl2:: natMX4	this work	JWP1,<br>SP1115
**SP1078**	h^- ade6-M216 leu1-32 ura4-D18 Tf2-2::lacZ(ura4$^+$)<br>dbl2:: natMX4	this work	JWP2,<br>SP1115
**SP1079**	h^- ade6-M216 leu1-32 ura4-D18 Tf2-2::lacZ(ura4$^+$)<br>dbl2:: natMX4	this work	JWP2,<br>SP1115
**SP1080**	h^- ade6-M216 leu1-32 ura4-D18 Tf2-2::lacZ(ura4$^+$)<br>dbl2:: natMX4	this work	JWP2,<br>SP1115
**SP1081**	h^- ade6-M216 leu1-32 ura4-D18 Tf2-3::lacZ(ura4$^+$)<br>dbl2:: natMX4	this work	JWP3,<br>SP1115
**SP1082**	h^- ade6-M216 leu1-32 ura4-D18 Tf2-3::lacZ(ura4$^+$)<br>dbl2:: natMX4	this work	JWP3,<br>SP1115
**SP1083**	h^- ade6-M216 leu1-32 ura4-D18 Tf2-3::lacZ(ura4$^+$)<br>dbl2:: natMX4	this work	JWP3,<br>SP1115
**SP1084**	h^- ade6-M216 leu1-32 ura4-D18 Tf2-4::lacZ(ura4$^+$)<br>dbl2:: natMX4	this work	JWP4,<br>SP1115
**SP1085**	h^- ade6-M216 leu1-32 ura4-D18 Tf2-4::lacZ(ura4$^+$)<br>dbl2:: natMX4	this work	JWP4,<br>SP1115
**SP1086**	h^- ade6-M216 leu1-32 ura4-D18 Tf2-4::lacZ(ura4$^+$)<br>dbl2:: natMX4	this work	JWP4,<br>SP1115
**SP1087**	h^- ade6-M216 leu1-32 ura4-D18 Tf2-5::lacZ(ura4$^+$)<br>dbl2:: natMX4	this work	JWP5,<br>SP1115
**SP1088**	h^- ade6-M216 leu1-32 ura4-D18 Tf2-5::lacZ(ura4$^+$)<br>dbl2:: natMX4	this work	JWP5,<br>SP1115
**SP1089**	h^- ade6-M216 leu1-32 ura4-D18 Tf2-5::lacZ(ura4$^+$)<br>dbl2:: natMX4	this work	JWP5,<br>SP1115
**SP1090**	h^- ade6-M216 leu1-32 ura4-D18 Tf2-6::lacZ(ura4$^+$)<br>dbl2:: natMX4	this work	JWP6,<br>SP1115
**SP1091**	h^- ade6-M216 leu1-32 ura4-D18 Tf2-6::lacZ(ura4$^+$)<br>dbl2:: natMX4	this work	JWP6,<br>SP1115
**SP1092**	h^- ade6-M216 leu1-32 ura4-D18 Tf2-6::lacZ(ura4$^+$)<br>dbl2:: natMX4	this work	JWP6,<br>SP1115
**SP1093**	h^- ade6-M216 leu1-32 ura4-D18 Tf2-7::lacZ(ura4$^+$)<br>dbl2:: natMX4	this work	JWP7,<br>SP1115
**SP1094**	h^- ade6-M216 leu1-32 ura4-D18 Tf2-7::lacZ(ura4$^+$)<br>dbl2:: natMX4	this work	JWP7,<br>SP1115
**SP1095**	h^- ade6-M216 leu1-32 ura4-D18 Tf2-7::lacZ(ura4$^+$)	this work	JWP7,

	dbl2:: natMX4		SP1115
**SP1096**	*h⁻ ade6-M216 leu1-32 ura4-D18 Tf2-8::lacZ(ura4⁺) dbl2:: natMX4*	this work	JWP8, SP1115
**SP1097**	*h⁻ ade6-M216 leu1-32 ura4-D18 Tf2-8::lacZ(ura4⁺) dbl2:: natMX4*	this work	JWP8, SP1115
**SP1098**	*h⁻ ade6-M216 leu1-32 ura4-D18 Tf2-8::lacZ(ura4⁺) dbl2:: natMX4*	this work	JWP8, SP1115
**SP1100**	*h⁻ ade6-M216 leu1-32 ura4-D18 Tf2-9::lacZ(ura4⁺) dbl2:: natMX4*	this work	JWP9, SP1115
**SP1101**	*h⁻ ade6-M216 leu1-32 ura4-D18 Tf2-9::lacZ(ura4⁺) dbl2:: natMX4*	this work	JWP9, SP1115
**SP1102**	*h⁻ ade6-M216 leu1-32 ura4-D18 Tf2-9::lacZ(ura4⁺) dbl2:: natMX4*	this work	JWP9, SP1115
**SP1103**	*h⁻ ade6-M216 leu1-32 ura4-D18 Tf2-10::lacZ(ura4⁺) dbl2:: natMX4*	this work	JWP10, SP1115
**SP1104**	*h⁻ ade6-M216 leu1-32 ura4-D18 Tf2-10::lacZ(ura4⁺) dbl2:: natMX4*	this work	JWP10, SP1115
**SP1105**	*h⁻ ade6-M216 leu1-32 ura4-D18 Tf2-10::lacZ(ura4⁺) dbl2:: natMX4*	this work	JWP10, SP1115
**SP1106**	*h⁻ ade6-M216 leu1-32 ura4-D18 Tf2-11::lacZ(ura4⁺) dbl2:: natMX4*	this work	JWP11, SP1115
**SP1107**	*h⁻ ade6-M216 leu1-32 ura4-D18 Tf2-11::lacZ(ura4⁺) dbl2:: natMX4*	this work	JWP11, SP1115
**SP1108**	*h⁻ ade6-M216 leu1-32 ura4-D18 Tf2-11::lacZ(ura4⁺) dbl2:: natMX4*	this work	JWP11, SP1115
**SP1109**	*h⁻ ade6-M216 leu1-32 ura4-D18 Tf2-12::lacZ(ura4⁺) dbl2:: natMX4*	this work	JWP12, SP1115
**SP1110**	*h⁻ ade6-M216 leu1-32 ura4-D18 Tf2-12::lacZ(ura4⁺) dbl2:: natMX4*	this work	JWP12, SP1115
**SP1111**	*h⁻ ade6-M216 leu1-32 ura4-D18 Tf2-12::lacZ(ura4⁺) dbl2:: natMX4*	this work	JWP12, SP1115
**SP1112**	*h⁻ ade6-M216 leu1-32 ura4-D18 Tf2-13::lacZ(ura4⁺) dbl2:: natMX4*	this work	JWP13, SP1115
**SP1113**	*h⁻ ade6-M216 leu1-32 ura4-D18 Tf2-13::lacZ(ura4⁺) dbl2:: natMX4*	this work	JWP13, SP1115
**SP1114**	*h⁻ ade6-M216 leu1-32 ura4-D18 Tf2-13::lacZ(ura4⁺) dbl2:: natMX4*	this work	JWP13, SP1115
**SP1115**	*h⁹⁰ leu1-32 ura4-D18 ade6-M210 dbl2::natMX4*	J. Gregan	JG17146

[a] omns: other auxotrophic markers not scored.

Table S2. List of oligonucleotide primers

Primer ID	5' - 3' sequence
**qSPBC32H8.12cfw**	GCAAGCGTGGTATTTTGACC
**qSPBC32H8.12crv**	CTTCTCACGGTTGGATTTGG
**qSPAC29E6.08fw**	CTGTCGTCTTGATCTCAAAACTAT
**qSPAC29E6.08rv**	AATTTAACATCGCAACTTCCTAC
**qSPBC3E7.02cfw**	GCGAAGTCGTCAATGAGAGA
**qSPBC3E7.02crv**	GGCAAGGTGACAGTCAATAGG
**qSPBPB21E7.07fw**	TTCGAGGTAATCCTGTTGCAGT
**qSPBPB21E7.07rv**	TCTGGGGTAAATATACGAAGTCTG
**qSPBC16E9.16cfw**	ACGTTGATTCTCGTCAACAGCT
**qSPBC16E9.16crv**	ATCATAGGCTTGAGTGGCACA
**qSPAC4H3.08fw**	AGACATTGCTCACAGGAGGAGA
**qSPAC4H3.08rv**	TTCACGTTCAATTAAATCTCTCGT
**qSPAC1786.02fw**	CGATGTTGAAGATTTTGTTCACG
**qSPAC1786.02rv**	GTGTGGTGGTCAACAGGAACGT
**qSPCC645.02fw**	GTCGAATAATCCCACATGCTACA
**qSPCC645.02rv**	CTTTGTCGAGAACCAAGGCA
**qSPAC186.05cfw**	GATCATAGGCTGTGAGCTTGGA
**qSPAC186.05crv**	CCCAACTAACACAGCAAACGA
**qSPBC336.01fw**	CAACGGTATTATTTTGGCAACA
**qSPBC336.01rv**	TGCAAGTACCTCGAAGATCCA
**qSPAC9.05fw**	TTTCTTCGTAACTCCTCAAACCA
**qSPAC9.05rv**	ATGAACAACTTCCACATAAGCGTA
**qcendgfw**	AAGGAATGTGCCTCGTCAAATT
**qcendgrv**	TGCTTCACGGTATTTTTTGAAATC
**qcendhfw**	GTATTTGGATTCCATCGGTACTATGG
**qcendhrv**	ACTACATCGACACAGAAAAGAAAACAA
**qTERRAfw**	GAAGTTCACTCAGTCATAATTAATTGGGTAACGGAG
**qTERRArv**	GGGCCCAATAGTGGGGGCATTGTATTTGTG
**qSPAC27D7.09Cfw**	GTAGCTTGTCCTGCTAGTGA
**qSPAC27D7.09Crv**	AAGTTGAAGTAGCAGCTGAA
**qSPCC965.11Cfw**	TTATCGTATGGCTTACCAAC
**qSPCC965.11Crv**	CAGATTCTCCAAACACTGAC
**qSPAC212.08Cfw**	GGACCTAGATTGGTTAACAC
**qSPAC212.08Crv**	TCGGTTGTGTTAATCCATAC
**qSPCC663.06cfw**	TTGTTAAGGAATTGAGCAAC
**qSPCC663.06crv**	TACCTACAGCTTTTGCAACT
**qSPAC869.07Cfw**	GTAATGGATGACTGTTGGAG
**qSPAC869.07Crv**	TATATTTCCCTGCACTTGAG
**qSPAC3G9.11cfw**	TTACAACGACATCAACACTG
**qSPAC3G9.11crv**	ATATGAACTTCAACGAGCTG
**qSPBPB2B2.08fw**	CTTTTCCGCGTATATTGTAG
**qSPBPB2B2.08rv**	CAAATTTTGTCCGAATACAT
**qSPAC15E1.02cfw**	AACTTACAGGCACTGTGACACTC
**qSPAC15E1.02crv**	ATCAAGGCAACACTTAAGCCTAC
**qSPBC947.05cfw**	CTTATAGGGTTTGCATTGCTCTT
**qSPBC947.05crv**	TGCTTTGTGAGTAGCCAAAAATA
**qSPCC737.04fw**	AAGAAGTTGGATGGTTGTTGTTT
**qSPCC737.04rv**	AAAATTTCACAGTCAACCGATCT
**qSPBC1773.06Cfw**	TGTAGGCTACCACCATGTTATTG
**qSPBC1773.06Crv**	TACCAACAAAGATACCACGAATG
**qSPAC11D3.01Cfw**	GAAAGAATATCTCGAAGAGCATGA
**qSPAC11D3.01Crv**	TCGAGGTTTTCTAACTCCTCTTG

**qSPAC186.01fw**	TGGTAATCCCTACTGCTGGTACT
**qSPAC186.01rv**	GAAGGGCTGATTTTATCATTGTC
**qSPCC736.02fw**	TCTCTATTCAACGTGGATTGCTA
**qSPCC736.02rv**	TTGGTATAAAACCTTCGTGTCCT

Figure S1

GAL4-BD **GAL4-AD** **SD-LW** **SD-LWH** **SD-LWA**

Empty vector Dbl2
Dbl2 Empty vector

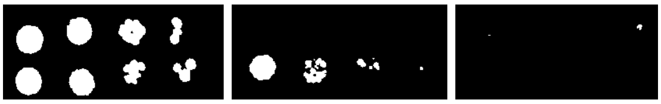

Figure S1. Dbl2 fused to the GAL4 DNA-binding domain is able to trigger expression from the reporter gene. Strains expressing Dbl2 fused to the GAL4 transcription activation domain or to the GAL4 DNA-binding domain were grown on SD plates lacking tryptophan and leucine (SD-L,W) and then spotted at 5-fold serial dilutions on SD plates lacking tryptophan and leucine (SD-L,W) or SD plates lacking tryptophan, leucine and histidine (SD-L,W,H) or SD plates lacking tryptophan, leucine and adenine (SD-L,W,A). The empty vectors pGADT7 and pGBKT7 containing GAL4 transcription activation domain and GAL4 DNA-binding domain, respectively were used as negative controls. Growth on plates without histidine indicates that Dbl2 is able to trigger expression from the reporter gene when fused to the GAL4 DNA-binding domain.

Figure S2

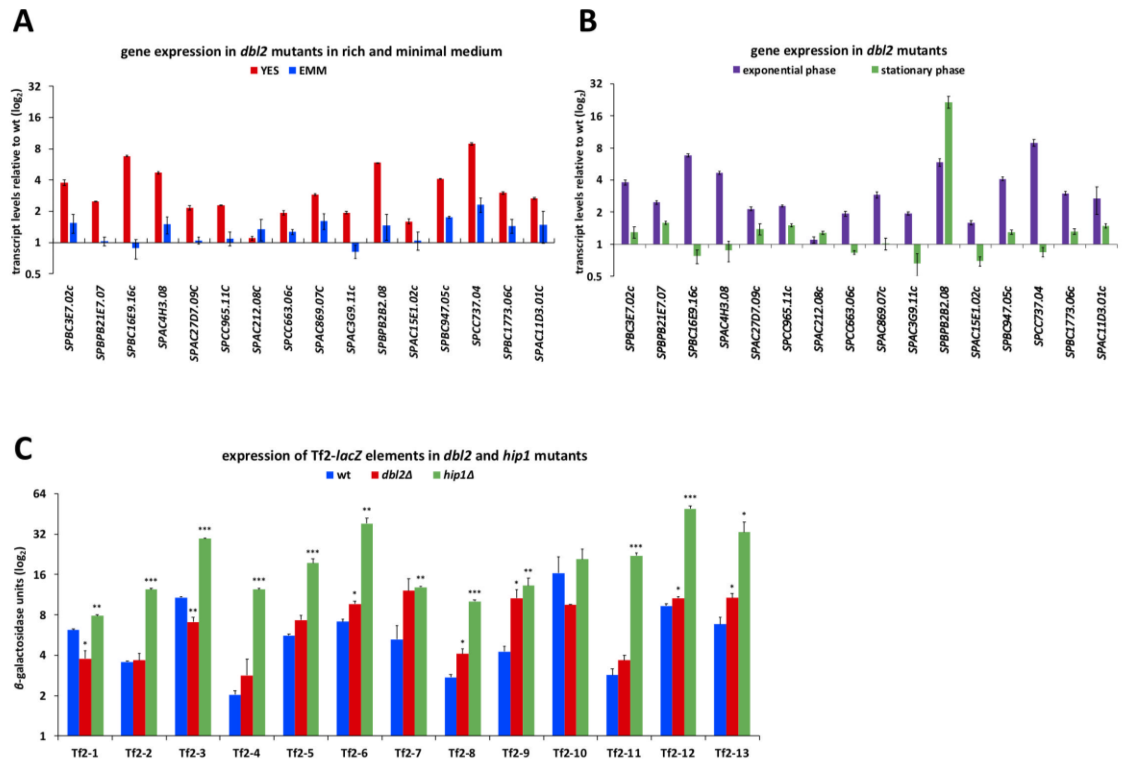

Figure S2. Deletion of *dbl2* affects expression of selected genes in minimal medium (A) and in YES medium at the stationary phase (B) to a lesser degree than in YES medium at the exponential phase. RNA was isolated from wild-type (SP065) and *dbl2Δ* (SP067) strains cultivated in YES and EMM2 medium until the exponential phase (OD_{595} = 0.5–0.55) or in YES until the exponential phase (OD_{595} = 0.5–0.55) and stationary phase (OD_{595} = 7–8). Expression of genes was measured using qPCR. The values plotted represent the mean from three independent biological replicates ± standard error of the mean. (C) Expression of Tf2-*lacZ* reporters in *dbl2Δ* was compared with the previously prepared reporters in wild-type and *hip1Δ* backgrounds (1). Strains were grown to OD_{595} = 0.75–0.8 in YES at 30°C, harvested and processed for liquid *β*-galactosidase assays. The values plotted represent the mean from three independent biological replicates ± standard error of the mean; asterisks denote $p < 0.05$ (*), $p < 0.01$ (**) and $p < 0.001$ (***) from two-tailed Student's *t*-tests, which was used to assess the significance of difference between the wild-type and mutant strains.

Figure S3

GO:MF		stats
Term name	Term ID	p_{adj}
urea transmembrane transporter activity	GO:0015204	1.281×10^{-2}
putrescine transmembrane transporter activity	GO:0015489	1.281×10^{-2}

GO:BP		stats
Term name	Term ID	p_{adj}
meiotic cell cycle	GO:0051321	7.215×10^{-4}
detoxification	GO:0098754	2.788×10^{-3}
cellular detoxification	GO:1990748	2.788×10^{-3}
cellular response to toxic substance	GO:0097237	5.603×10^{-3}
response to toxic substance	GO:0009636	7.301×10^{-3}
reproductive process	GO:0022414	8.615×10^{-3}
reproduction	GO:0000003	9.859×10^{-3}
one-carbon compound transport	GO:0019755	2.652×10^{-2}
urea transport	GO:0015840	4.993×10^{-2}
putrescine transport	GO:0015847	4.993×10^{-2}
urea transmembrane transport	GO:0071918	4.993×10^{-2}

GO:CC		stats
Term name	Term ID	p_{adj}
plasma membrane	GO:0005886	4.982×10^{-2}

Figure S3. GO enrichment analysis of protein-coding genes upregulated in the *dbl2Δ* mutant. Functional enrichment analysis was performed using g:Profiler (version e94_eg41_p11_6f51822) with g:SCS multiple testing correction method applying significance threshold of 0.05. GO – gene ontology, MF – molecular function, BP – biological process, CC – cellular compartment.

Figure S4

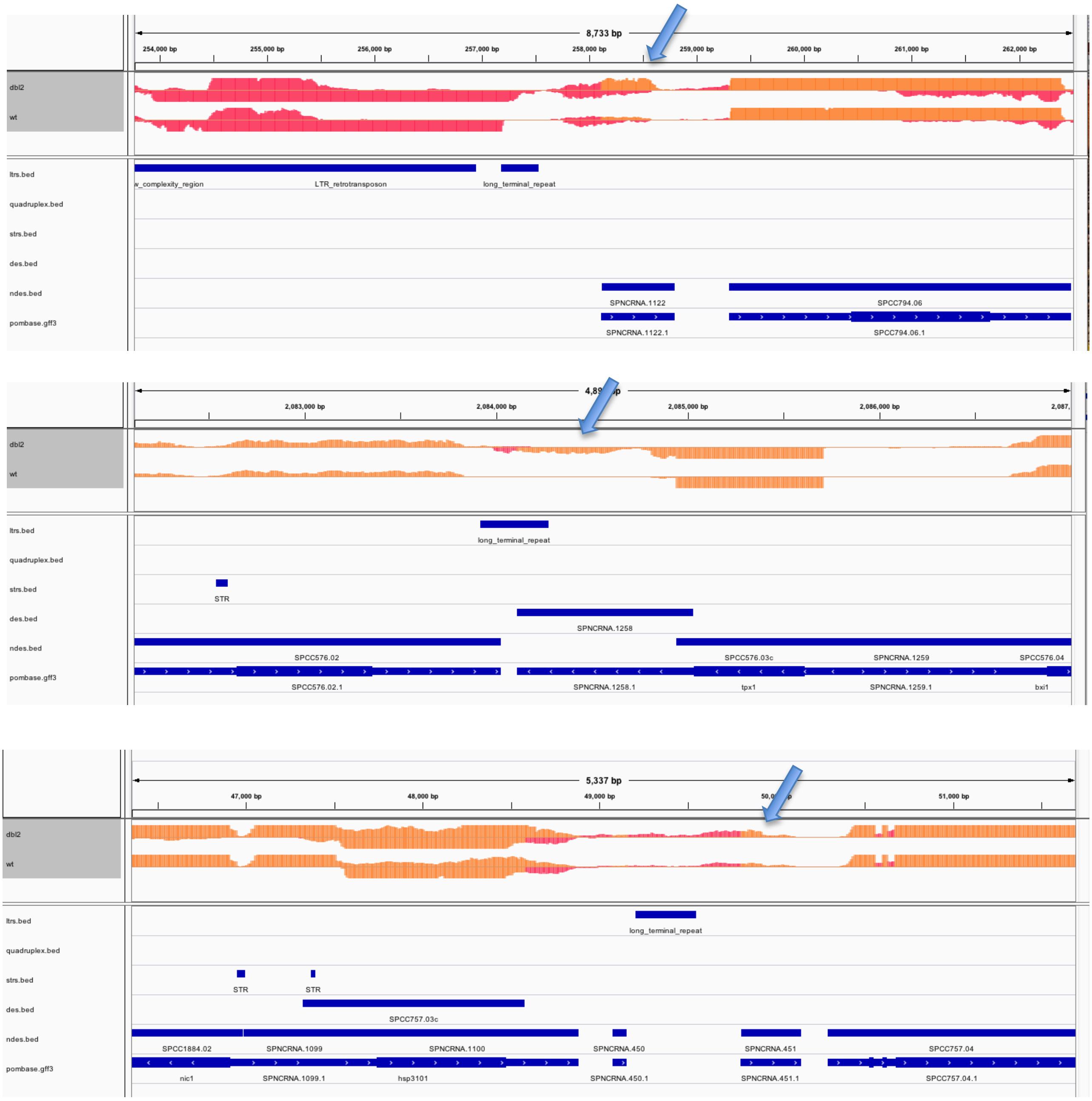

Figure S4. Visual examples of repeat-containing genomic regions created by the Integrative Genomics Viewer (IGV). IGV displays data in horizontal rows. RNA-seq data (average from 4 biological replicates) from *dbl2Δ* and wt on forward and reverse strand are represented as orange and red lines on the upper and lower part of the plot, respectively. The horizontal thin line for each strain separates the forward from the reverse strand. Orange lines represent transcripts generated from annotated genes and red lines represent transcripts originating outside of annotated genes. This unusual transcription occurring outside of annotated genes can initiate in almost any genomic context and has been described as ‘pervasive’ (2). Pervasive transcripts can be either sense or antisense with respect to the overlapping gene. Repeat-adjacent genes were upregulated in the *dbl2Δ* mutant. The top row indicates which portion of the chromosome is displayed. Track names are listed in the far left panel. Genes and other

genomic features, such as long terminal repeats and short tandem repeats (STRs) are displayed in tracks. Light blue arrows mark upregulated regions in the *dbl2Δ* mutant adjacent to LTRs.

Figure S5

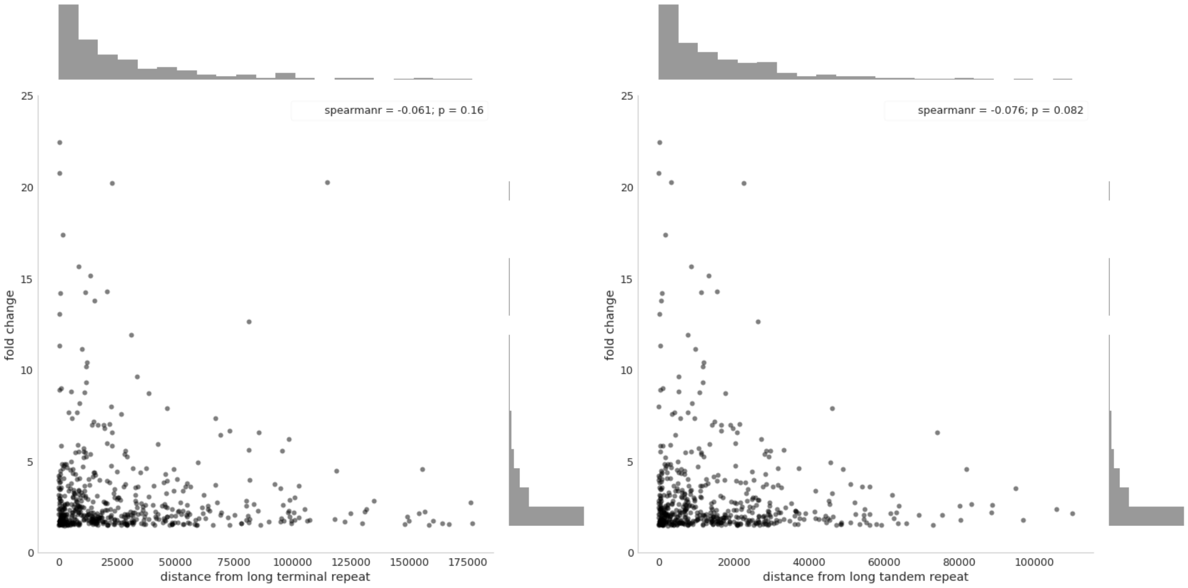

Figure S5. The scatter plots show only a very small correlation between the transcript fold change and the distance from a long terminal or a long tandem repeat (p = 0.16 and p = 0.082, respectively). Two genes with overly high fold change (36.2-fold increase and 53.7-fold increase) were excluded.

Figure S6

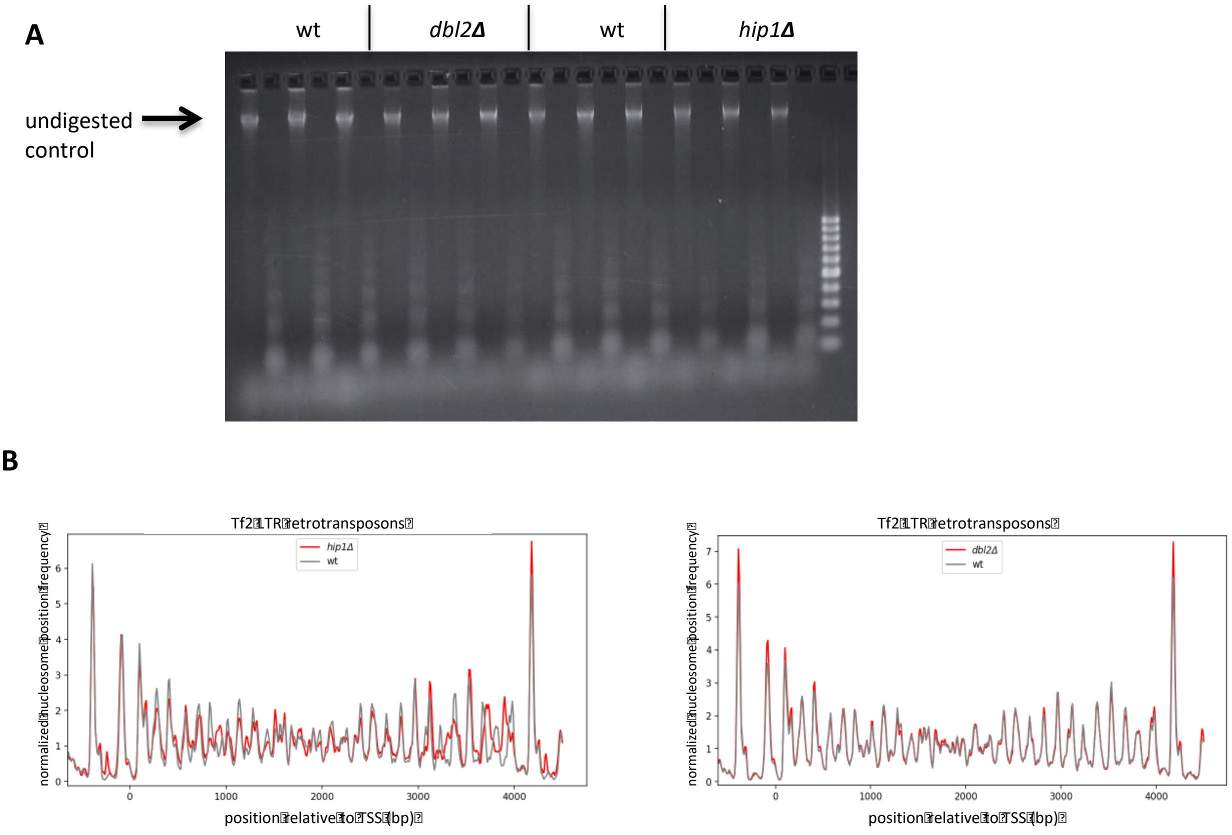

Figure S6.

(A) Ethidium bromide-stained gel separation of DNA pools extracted from MNase digested *S. pombe* chromatin used for sequencing. For each strain three digested biological replicates and three undigested controls were prepared. M stands for GeneRuler 100 bp DNA ladder.

(B) Average nucleosome occupancy profiles of Tf2 elements aligned at the transcription start site (TSS) in wild-type (SP072), *hip1Δ* (SP456), wild-type (SP065) and *dbl2Δ* (SP067) strains. The plotted values are the mean of three independent biological replicates.

Figure S7

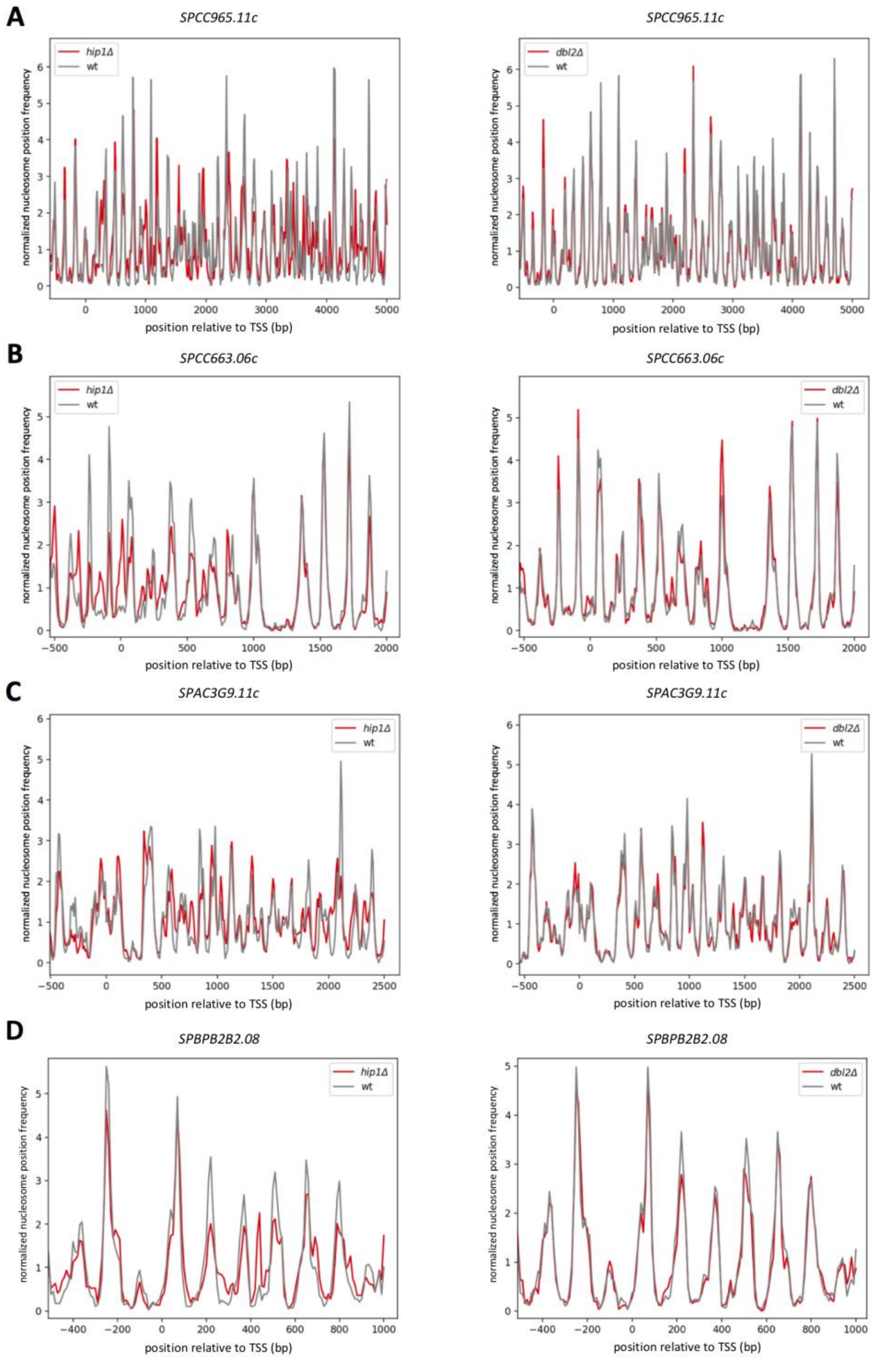
A
SPCC965.11c
hip1Δ
wt
dbl2Δ
normalized nucleosome position frequency
position relative to TSS (bp)
B
SPCC663.06c
C
SPAC3G9.11c
D
SPBPB2B2.08

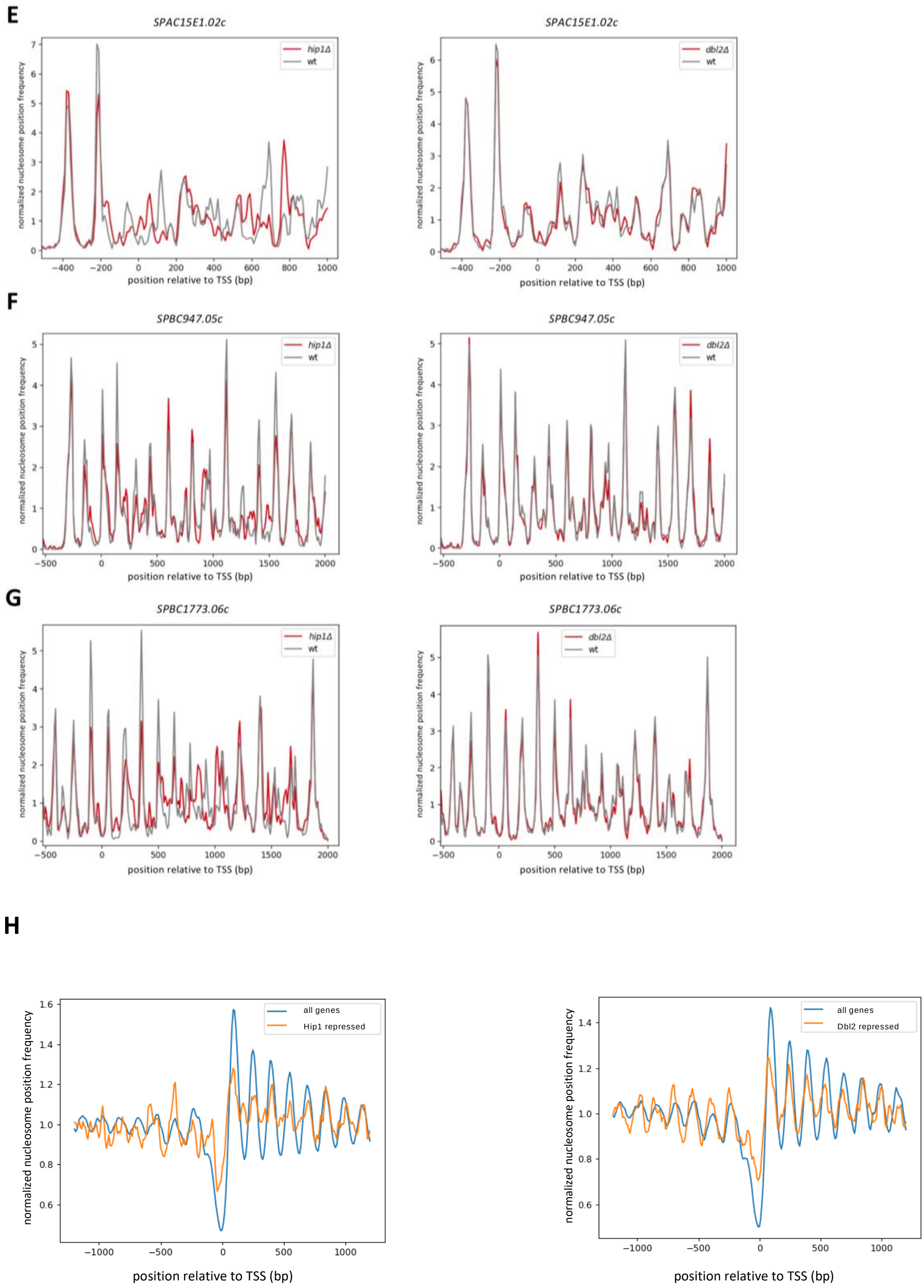

Figure S7.

(A-G) Nucleosome occupancies of individual genes relative to the TSS in the *hip1Δ* (SP456) and *dbl2Δ* (SP67) mutants and wild-types (SP065, SP072). The values plotted represent the mean from three independent biological replicates.

(H) Average nucleosome profiles for all genes aligned at the TSS compared with the nucleosome profile of a set of HIRA (1) and Dbl2-repressed genes. The values plotted represent the mean from three independent biological replicates.

Figure S8

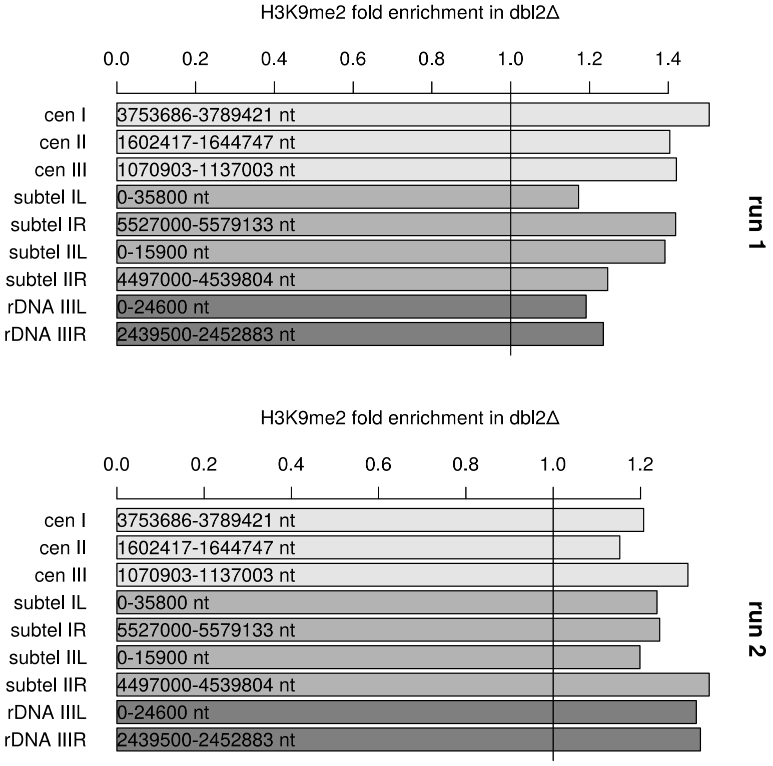

Figure S8. H3K9me2 occupancy at subtelomeres, centromeres and rDNA is increased in the *dbl2Δ* mutant. H3K9me2 ChIP-seq coverage in each sample was first normalised to the corresponding total H3 ChIP-seq coverage, and the *dbl2Δ* values were then further normalised to the WT values from the corresponding biological replicate. Final normalised H3K9me2 occupancy is shown as fold enrichment at major heterochromatin loci from two independent experiments. The subtelomeres are here defined as regions of H3K9me2 enrichment in wild-type cells.

Figure S9

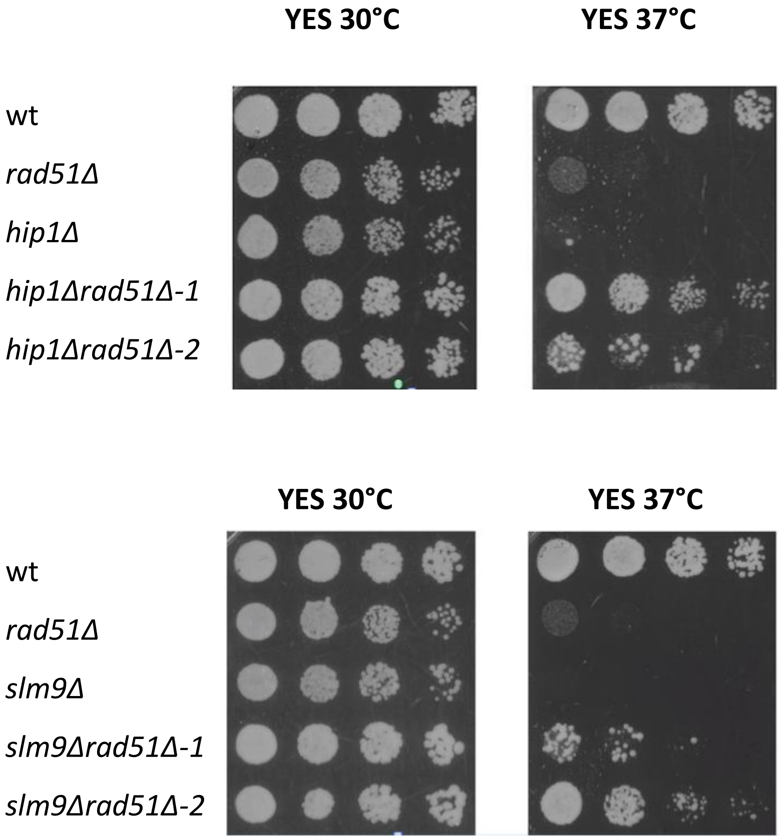

Figure S9. Combining mutations in *hip1Δ* or *slm9Δ* with *rad51Δ* partially rescued the growth at 37°C. Wild-type (SP065) and mutant strains *rad51Δ* (SP068), *hip1Δ* (SP456), *hip1Δrad51Δ* (SP792, SP793), *slm9Δ* (SP462) and *slm9Δrad51Δ* (SP796, SP797) were cultivated until the exponential phase in YES medium. Tenfold serial dilution of cell suspensions was spotted on the indicated plates. Images were taken after 3-day cultivation at 30°C or 37°C.

References

1. Anderson,H.E., Wardle,J., Korkut,S. V., Murton,H.E., Lopez-Maury,L., Bahler,J. and Whitehall,S.K. (2009) The Fission Yeast HIRA Histone Chaperone Is Required for Promoter Silencing and the Suppression of Cryptic Antisense Transcripts. *Mol. Cell. Biol.*, **29**, 5158–5167. https://doi.org/10.1128/MCB.00698-09

2. Kapranov, P., Cheng, J., Dike, S., Nix, D.A., Duttagupta, R., Willingham, A.T., Stadler, P.F., Hertel, J., Hackermuller, J., Hofacker, I.L., Bell, I., Cheung, E., Drenkow, J., Dumais, E., Patel, S., Helt, G., Ganesh, M., Ghosh, S., Piccolboni, A., Sementchenko, V., Tammana, H., Gingeras, T. R. (2007) RNA maps reveal new RNA classes and a possible function for pervasive transcription. *Science*, **316**, 1484-1488. http://doi.org/10.1126/science.1138341
